# Rock, Paper, Scissors: Harnessing Complementarity in Ortholog Detection Methods Improves Comparative Genomic Inference

**DOI:** 10.1534/g3.115.017095

**Published:** 2015-02-23

**Authors:** M. Cyrus Maher, Ryan D. Hernandez

**Affiliations:** * Department of Epidemiology and Biostatistics, University of California, San Francisco, University of California, San Francisco, San Francisco, California; † Department of Bioengineering and Therapeutic Sciences, University of California, San Francisco, San Francisco, California; ‡ Institute for Human Genetics, University of California, San Francisco, San Francisco, California; § Institute for Quantitative Biosciences (QB3), University of California, San Francisco, San Francisco, California 94158

**Keywords:** multiple sequence alignment, ortholog detection, comparative genomics, positive selection, open source software

## Abstract

Ortholog detection (OD) is a lynchpin of most statistical methods in comparative genomics. This task involves accurately identifying genes across species that descend from a common ancestral sequence. OD methods comprise a wide variety of approaches, each with their own benefits and costs under a variety of evolutionary and practical scenarios. In this article, we examine the proteomes of ten mammals by using four methodologically distinct, rigorously filtered OD methods. In head-to-head comparisons, we find that these algorithms significantly outperform one another for 38–45% of the genes analyzed. We leverage this high complementarity through the development MOSAIC, or **M**ultiple **O**rthologous **S**equence **A**nalysis and **I**ntegration by **C**luster optimization, the first tool for integrating methodologically diverse OD methods. Relative to the four methods examined, MOSAIC more than *quintuples* the number of alignments for which all species are present while simultaneously maintaining or improving functional-, phylogenetic-, and sequence identity-based measures of ortholog quality. Further, this improvement in alignment quality yields more confidently aligned sites and higher levels of overall conservation, while simultaneously detecting of up to 180% more positively selected sites. We close by highlighting a MOSAIC-specific positively selected sites near the active site of TPSAB1, an enzyme linked to asthma, heart disease, and irritable bowel disease. MOSAIC alignments, source code, and full documentation are available at http://pythonhosted.org/bio-MOSAIC.

Orthologs are genes that derive from a common ancestral gene but that have diverged from one another through speciation. This route is in contrast to paralogs, which arise through gene duplication within a given genome. It is common in comparative genomics and phylogenetics to extract evolutionary information about a particular gene from its alignment with orthologous sequences. To enable this analysis, orthologs must first be inferred, making ortholog detection (OD) an indispensible first step in a variety of phylogenetic inference tasks (Ciccarelli *et al.* 2006; Yandell and Ence 2012).

In general, existing OD methods can be classified as tree-based, graph-based, or a hybrid of the two (Altenhoff and Dessimoz 2012). Tree-based methods may use reconciliation techniques between gene and species trees or may rely on the gene tree alone. Graph-based methods can use a variety of metrics to quantify similarity between sequences, including Basic Local Alignment Search Tool (BLAST) scores or sequence identity. Information about the conserved gene neighborhood also may be included in this context. Techniques such as Markov clustering may then be applied to create orthologous groups, or one may simply define clusters based on a graph’s existing connections (Kuzniar *et al.* 2008).

Unfortunately, the few benchmarking studies that have sampled broadly from this methodologic diversity have provided equivocal results. Although there are general patterns in relative effectiveness between methods, performance is highly context-dependent and does not always favor more sophisticated approaches (Hulsen *et al.* 2006; Chen *et al.* 2007; Altenhoff and Dessimoz 2009a). This is discouraging from the point of view of identifying a single best OD method, but it also suggests a new and relatively facile avenue for methodologic improvement. By harnessing differences between OD methods, a wide variety of algorithms may play complementary roles within a cooperative inference framework.

We begin our analysis with a comprehensive comparison of four popular and methodologically distinct OD methods: (1) MultiParanoid, a reciprocal-BLAST plus Markov clustering method (Alexeyenko *et al.* 2006); (2) TBA, a synteny-based aligner used to produce University of California Santa Cruz’s MultiZ alignments (Blanchette *et al.* 2004); (3) six-frame translated BLAT, a fast, approximately-scored protein query approach that does not rely on predicted proteomes (Kent 2002); and (4) OMA, a well-established tree-graph hybrid method (Altenhoff *et al.* 2011). Applying these methods to OD in a range of primates and closely related mammals, we demonstrate that methodological performance varies widely by species and appears to depend critically on genome quality.

Next, we characterize the striking performance gains yielded by combining these methods. This is demonstrated using sequence identity, phylogenetic tree concordance, and hidden Markov model-based functional agreement. This improvement in alignment quality translates to higher estimated levels of overall conservation. At the same time, we detect up to 180% more positively selected sites. We close by highlighting a novel positively selected site (PSS) near the active site of TPSAB1, an enzyme linked to asthma and irritable bowel disease.

The implementation of this novel approach for the integration of diverse ortholog detection methods is presented as the software tool, MOSAIC, or **M**ultiple **O**rthologous **S**equence **A**nalysis and **I**ntegration by **C**luster optimization. MOSAIC is implemented as a well-documented python package that can be installed using easy_install bio-mosaic from the command-line. MOSAIC alignments, source code, and full documentation are available at http://pythonhosted.org/bio-MOSAIC.

## Materials and Methods

### Retrieval of orthologs

For each human consensus coding sequence (version GRCh37.p9), we sought to retrieve orthologs for chimp, gorilla, orangutan, rhesus macaque, marmoset, bushbaby, cat, cow, and horse using four methodologically distinct methods: (1) MultiParanoid (Alexeyenko *et al.* 2006); (2) TBA (Blanchette *et al.* 2004); (3) six-frame translated BLAT (Kent 2002); and (4) OMA (Altenhoff *et al.* 2011; July 2013 release). We used MultiParanoid over OrthoMCL (Li *et al.* 2003) because the latter produced no errors or output after careful execution of the thirteen-stage analysis protocol. For all methods, genomic data were retrieved for the genome builds listed in Table 1.

**Table 1:** Genome builds

Genome	Version	Release
Chimp	panTro4	Feb-11
Gorilla	gorGor3.1	May-10
Orangutan	ponAbe2	Jul-11
Rhesus macaque	rheMac3	Oct-10
Marmoset	calJac3	Mar-09
Bushbaby	otoGar3	May-11
Cat	felCat5	Sep-11
Cow	bosTau7	Oct-11
Horse	equCab2	Sep-07

For MultiParanoid (Alexeyenko *et al.* 2006), an all-*vs.*-all blast search was run using the following command structure:

blastp -db $blastdatabase -query [query file] -out [output file] -evalue .01 -num_threads [number of threads] -outfmt 6 -db_soft_mask 21 -word_size 3 -use_sw_tback

From this output, ortholog predictions were produced using the standard MultiParanoid protocol.

For BLAT (Kent 2002), genomes for each species of interest were downloaded from the NCBI Entrez Genome database (McEntyre and Ostell 2002). Queries were conducted using the following command structure:

blat -q=prot -t=dnax -minIdentity=70 –extendThroughN [genome file] [query file] [output file]

In the case of MultiZ (Blanchette *et al.* 2004), CCDS orthologs were downloaded directly from the UCSC genome browser (Kent *et al.* 2002). For OMA (Altenhoff *et al.* 2011), ortholog predictions were downloaded from omabrowser.org (December 2012 release). For genes with more than one CCDS, orthologs were mapped to each analyzed transcript. Finally, ortholog predictions from metaPhOrs (Pryszcz *et al.* 2011) were retrieved from release v201009 (June 2012).

### Filtering putatively non-orthologous sequences

All ortholog detection methods produce false positives. For example, this can result when a gene deletion on one lineage means that no true ortholog exists in a given species. Typically, these issues are dealt with through rigorous filtering of input alignments. The intuition is that by applying a stringent sequence similarity filter, we can remove the vast majority of evolutionarily unrelated genes. We use this filtering approach to ensure that only credible, putatively orthologous sequences are included in the analysis. Because of heterogeneity in genome quality, similarity cutoffs were chosen heuristically, considering the known level of genome-wide divergence between human and the species of interest, as well as the overall distributions of percent identity between putative orthologs in the two species. Specifically, we first chose a cutoff based on the species-specific levels of percent identity to human. We then updated these numbers based on spot checks of borderline alignment cases. These cutoffs were as follows: chimp: 82%, gorilla: 77%, orangutan: 75%, rhesus macaque: 73%. A cutoff of 70% was employed for marmoset, bushbaby, cat, cow, and horse. For applications where consistency across methods is not important, these cutoffs could be chosen using downstream quality metrics such as those presented in Figure 4. Note that such an approach would still require the user to specify a tradeoff between the quality and number of orthologs.

## MOSAIC: OD integration as cluster optimization

**Figure 1:**
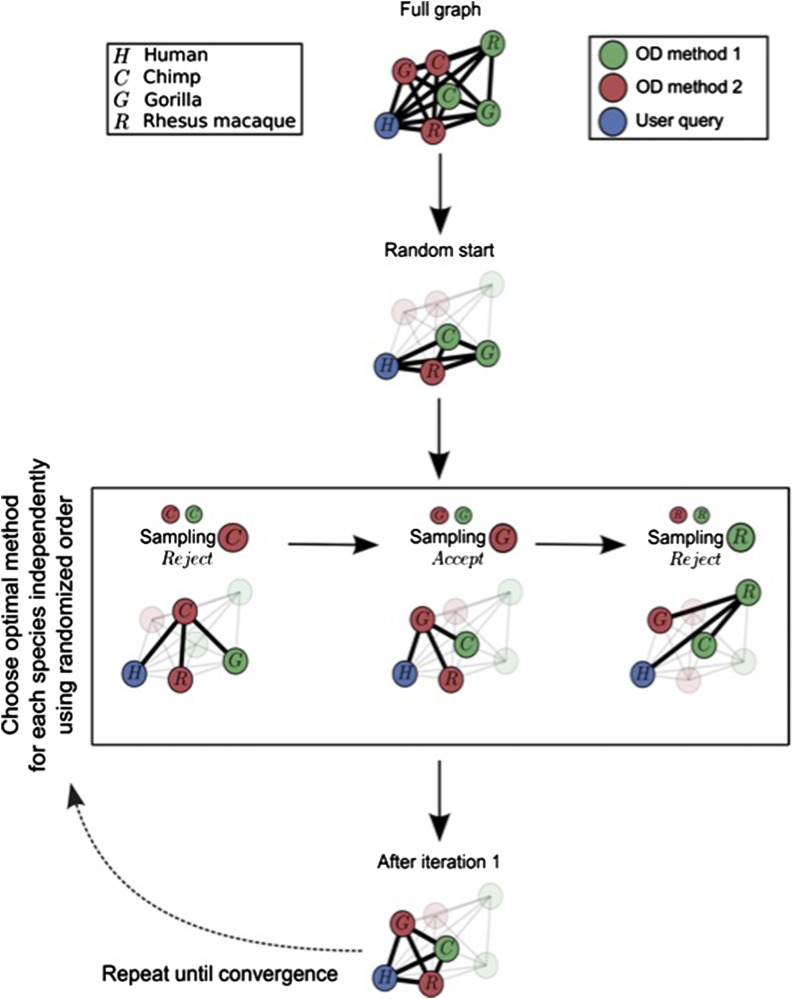
A schematic of the sequence selection algorithm. Steps: (1) Construct graph; (2) Choose the sequence from a random OD method for each species; (3) Iterate through species. For each species, pick the orthologs with highest similarity to the current best choices for all other species; (4) Return current best choices if no changes are made after iterating through all species; (5) To find global optimum, repeat steps 1-4 with random sampling paths.

MOSAIC provides a highly flexible, graph-based framework for integrating diverse OD methods. For a given reference sequence, proposal orthologs are conceptualized as nodes in a graph, connected with edges weighted according to the pairwise similarity between sequences (Figure 1). The task of OD integration is then to choose proposal orthologs from each species such that a chosen measure of intra-cluster similarity is optimized.

### MOSAIC optimizes (weighted) pairwise similarities

To begin, MOSAIC calculates pairwise similarities between all orthologs from different species. Percent identity- and BLAST-based similarity metrics are provided by default, but user-defined similarity metrics are also accepted. These similarity scores define edge weights, which are used to construct a graph such as the one presented at the top of Figure 1. Once this full graph is constructed, it is highly recommended that it be quality filtered using user-specified similarity cutoffs. This step is necessary to minimize the effect of gene loss, duplication, etc. Once the graph is cleaned, MOSAIC then chooses at most one proposal ortholog from each species so that the overall pairwise similarity between accepted sequences is optimized.

To accommodate user priorities, pairwise similarities can be weighted such that sequences from different species contribute unequally to the total similarity score. For uniform weights, this is equivalent to maximizing the average pairwise similarity. In the case where only similarity to a reference sequence is of interest, this reduces to simply accepting the ortholog for each species that is most similar to the reference.

### Optimization is carried out using cyclic coordinate descent

For *m* OD methods and *s* species, there are up to *m^s^* possible integrated alignments. In the case analyzed in this paper, *m = 4* and *s = 10*. This translates to over a million possible integrated alignments for each of the ~25,000 reference sequences considered. It is clear to see from this example that an exhaustive optimization becomes quickly infeasible. Therefore, MOSAIC choses optimal clusters using cyclic coordinate descent (CCD), an efficient non-derivative optimization algorithm (Bertsekas 1999).

In Figure 1, we illustrate the way CCD functions in the context of MOSAIC. After the full graph that includes all orthologous sequences is built, random orthologs from each species are chosen as the current best. MOSAIC then loops through the species of interest in a random order. For each species, MOSAIC chooses the sequence that optimizes cluster tightness, given the current best sequences for all other species. This process is repeated until no further improvements can be made to cluster tightness. Finally, because CCD is prone to finding local rather than global optima, this entire process is repeated multiple times with random starting points and sampling paths.

### Scoring and optimization procedures for this study

For the alignments presented here, we consider a protein set with relatively low levels of evolutionary divergence. We chose percent identity as our metric for sequence divergence. Note that several other popular scoring functions are implemented in MOSAIC. For more distantly related species, the application of scoring matrices (Dayhoff *et al.* 1978; Henikoff 1992) or Hidden Markov Models (Ebersberger *et al.* 2009) may be preferable. To reduce computational costs related to pairwise alignment, we considered only similarities between orthologs and the human target sequence. The optimization procedure was then equivalent to choosing, for each species, the ortholog among all methods that is most similar to the human sequence. This approach corresponds to the arguments edgefunc=’perID’, optrule=’pairwise’ when calling the Mosaic constructor in mosaic.py (see: http://pythonhosted.org/bio-MOSAIC/Module.html).

### Example: measuring similarity

Percent identity was calculated as the percent of sites in the human sequence that were identical in the orthologous sequence. For example, the hypothetical sequence below would be scored as 71% identical (5/7), because there are two mismatches between the seven sites present in the human sequence and the character to which those sites are aligned in the chimp sequence (sites where the human sequence has been deleted or the outgroup has an insertion are ignored):

Human A W V A - T F D
Chimp - W V R Y T F D

### A note on gene loss, duplication, and divergent evolution

For any query protein, there is a risk that a related gene in another genome has been deleted, or has not maintained the same function and so provides inapplicable evolutionary information. In the case of deletions, it is unlikely that a non-homologous gene would be suitably similar to be classified as an ortholog. Divergence in function would be expected to sharply increase sequence divergence. In many cases such functionally distinct can be removed by suitably stringent sequence similarity filters.

Another pitfall in ortholog detecion is gene duplication within a particular lineage. This results in so-called in-paralogs, which may inject additional bias if, compared to the query protein, the most functionally similar of the set is not the most similar at the sequence level. Although this is possible, it is the exception and not the rule under reasonable models of evolution. Indeed, experimental data from several model systems has demonstrated that there is an extremely high correlation between functional conservation and sequence conservation (Mashiyama *et al.* 2014; Zhao *et al.* 2014). Taking the single most similar sequence from a paralogous group is therefore a rational and effective (albeit imperfect) approach to this problem. For this reason, MOSAIC does not exclude putatively orthologous sequences that have paralogs in the source genomes. It rather picks the paralogous sequence that is most likely to share the same function as the other putatively orthologous sequences under examination. We will show that this decision allows us to capture more putative orthologs while simultaneously improving ortholog quality by all commonly used metrics.

In summary, MOSAIC is adapted to producing multiple sequence alignments (MSAs) that are functionally informative at the site-level. For other applications, researchers may wish to infer genomic events such as gene loss, duplication, horizontal gene transfer, and/or incomplete lineage sorting (*e.g.*, Capra *et al.* 2013). This involves jointly examining functionally diverged paralogous groups alongside their corresponding orthologs. This task generally requires a combination of tools such as MultiParanoid (to infer paralogs; Remm *et al.* 2001), RaxML (to build gene and species trees; Stamatakis 2014), and Notung (to reconcile gene trees with species trees and infer evolutionary events; Stolzer *et al.* 2012). For applications such as this, MOSAIC alignments can still be leveraged to help ensure the inclusion of relevant sequences. Likewise, reconstructed evolutionary histories can be used to flag, among tens of thousands of automatically generated MOSAIC alignments, those exceptional cases that could benefit most from manual inspection.

### Multiple sequence alignment

Retrieved sequences were jointly aligned to query proteins using MSAprobs (Liu *et al.* 2010), a multithreaded aligner with better performance benchmarks than many top aligners, including ClustalW, MAFFT, MUSCLE, ProbCons, and Probalign (Liu *et al.* 2010). Importantly, MSAprobs has the further advantage of providing, for each column of an alignment, dependable estimates of the confidence of the alignment at the site.

### Quality assessment

One approach to evaluating the performance of ortholog detection methods is to restrict analysis to validation sets, which usually consist of small, curated gene groups from unicellular model organisms (*e.g.*, Salichos and Rokas 2011). Known true positive and true negative relationships allow researchers to calculation power and sensitivity, and even provide the possibility of applying supervised learning techniques such as support vector machines or random forest classifiers. Although such results are assuredly internally valid, it is unclear whether these results generalize beyond this small and highly biased subset of genes. As a simple example, “true” orthologous relationships often are restricted to cases where synteny is also maintained. Filtering out nonsyntenic orthologous sequences will thus significantly bias performance metrics toward OD methods that use syntenic information. Furthermore, this approach to quality assessment does not allow researchers to evaluate performance on their own arbitrary dataset. It is for these reasons that we decided to use the quality metrics described below. Strictly speaking, the details necessary to truly establish orthology are buried deep in evolutionary time. We therefore believe that ortholog detection is perhaps better viewed as an unsupervised learning problem that is amenable to graph-based cluster optimization.

### Sequence identity

MOSAIC optimizes pairwise sequence similarity. In this example, sequence identity is used as the similarity measure, and pairwise similarities are weighted such that only concordance with the human reference sequence is considered. To achieve greater separation between metrics used for optimization and assessment, comparisons of sequence identity were performed in the context of the full MSAs. We believe this choice is sensible because it is the quality of the MSA that is of primary importance to many downstream phylogenetic inference tasks. In addition, this approach allows us to indirectly incorporate information about intra-cluster similarity. As an MSA incorporates increasingly divergent sequences, performance relative to pairwise alignments is expected to progressively degrade.

### Tree concordance

For each MSA, gene trees were built using RAxML (Stamatakis and Alachiotis 2010). An unweighted Robinson-Foulds (RF) distance (Robinson and Foulds 1981) was then calculated between each gene tree and the known species tree using the python module dendropy (Sukumaran and Holder 2010). Briefly, the unweighted RF distance counts the number of operations required to transform one tree into the other. This quantity is equal to the total number of splits that are present in one tree but not the other. To normalize for variations in tree size, we then divided this distance by the sum of the total number of splits in the gene and species trees (Yu *et al.* 2011). To summarize the genome-wide distribution of normalized RF distances, we took the area under the curve of the cumulative distribution function. This was limited to values less than 0.4, since beyond this value there is little difference between the observed curves (see Supporting Information, Figure S5). This metric is superior to, *e.g.*, calculating the proportion of genes below a given threshold because it up-weights smaller RF distances as opposed to, in effect, using non-zero uniform weights below the cutoff value.

### Functional concordance

Profile HMMs were downloaded from the PfamA protein families database (Punta *et al.* 2012). Each sequence was then annotated using the top scoring functional class retrieved by querying that sequence against the database of all PfamA protein family HMMs. This search was conducted using HMMER3 (Eddy 2011). Functional concordance was then measured as a binary quantity, corresponding to whether or not a putative orthologous sequence had the same inferred function as its cognate human sequence. It is important to note that not all PfamA HMMs are functionally validated. In cases where experimental validation is unavailable, these HMMs provide a family-specific scoring function that nevertheless yields information not contained in naïve sequence identity measures.

### Evolutionary analysis

#### Gene-level conservation:

Alignments were analyzed using phylogenetic analysis by maximum likelihood (PAML) (Yang 2007). For each alignment three models were fit: (1) a neutral model where the ration of the non-synonymous to synonymous substitution rates (dN/dS) is fixed at one, (2) a conservation model where dN/dS is less than or equal to one, and (3) a positive selection model where some fraction of the sequence is fit under the conservation model, while another dN/dS parameter is estimated freely for the remainder of the sequence. Because evolutionary models are not in general nested, we performed model selection via the popular Akaike information criterion, a method that penalizes a model’s fit by its number of included variables (Akaike 1973) and is asymptotically equivalent to maximizing the model’s predictive performance on unseen data (Stone 1977).

Despite rigorous model selection procedures, in rare cases PAML may estimate very high levels of selection over a tiny proportion of a given sequence (even a single site), leading to greatly inflated average levels of dN/dS. To reduce the influence of outlying estimates of selection, all dN/dS values greater than 3 were excluded for the analysis. For all methods, this corresponded to less than 0.05% of all sequences.

#### Site-level positive selection:

The program sitewise likelihood ratio (SLR) (Massingham and Goldman 2005) was used to estimate the number of positively selected sites in each sequence. To eliminate false positives caused by poorly aligned sites, we filtered out all sites estimated by MSAprobs to be aligned to less than 95% confidence. All included positively selected sites estimated at 95% confidence or greater by SLR were included in the subsequence comparison.

To assess agreement in PSS, we calculated the degree of overlap between PSS from all pairs of methods. This was calculated as the size of the genome-wide intersection between sites divided by the union of said sites.

### Mapping positively selected sites onto three-dimensional structures

We leveraged UniProt mapping files (http://www.uniprot.org/docs/pdbtosp; accessed 9/30/14) to determine which proteins had a relevant structure in the Protein Data Bank (PDB; Berman 2000). We then aligned sequences between PDB structures and candidate genes to determine the degree of coverage and to obtain a mapping between residues. We found 2003 genes for which there was a structure with greater than 70% coverage. Of these, 787 had PSS results from all five ortholog detection methods from at least one species. Reasons for missing data comprise more than 4 missing species in source MSAs and lack of convergence in the PSS calculation. Within this set of 787 genes, 76 proteins had PSS from MOSAIC that were not found with any of the component methods. From this point, the example of TPSAB1 was quickly identified by manual inspection. We then downloaded PDB structure 2ZEC to visualize the location of positively selected sites. To validate sequences used in the analysis, we blasted each ortholog against the human SwissProt database. TPSAB1 was the most similar human protein in each case, confirming that we had retrieved best bidirectional hits. Annotations for each protein were also consistent with alpha/beta tryptases activity (Table S1).

## Results and Discussion

### Ortholog detection methods frequently outperform one another

**Figure 2:**
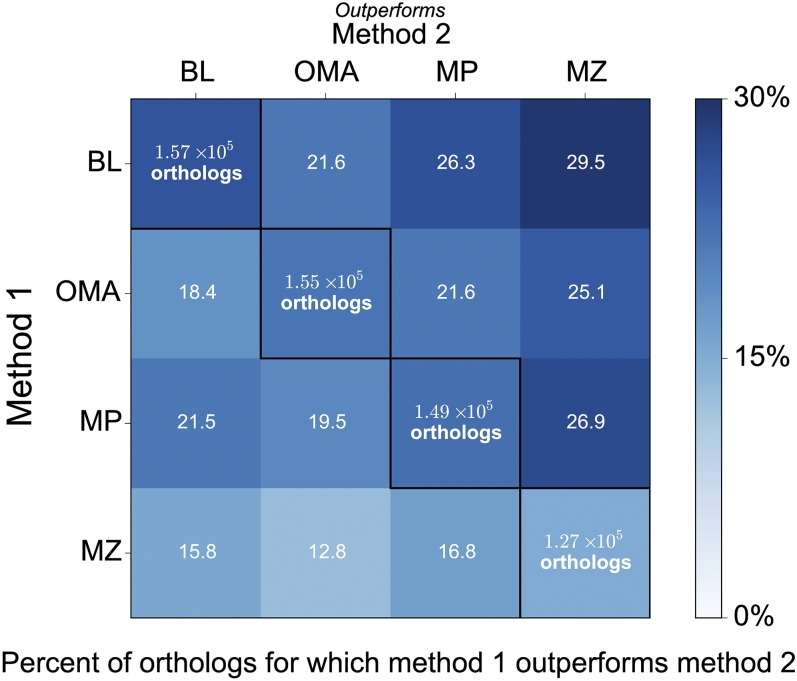
Comparison of sequence identity levels between methods. Heat map of the percent of orthologs for which MultiParanoid (MP), OMA (OMA), BLAT (BL), and MultiZ (MZ) outperform one another. Performance is based on percent identity of each method’s orthologs to the human sequence. One method is considered to outperform another method if it improves percent identity by at least five percentage points. Text in diagonal cells shows the number of orthologs identified by each method, colored by the percent of orthologs for which a given method outperforms all the others.

We begin with a comprehensive comparison of four popular, methodologically distinct OD methods. In Figure 2, we show the head-to-head performances of these different methods for a range of primates and closely related mammals. Performance is assessed using alignments between all human consensus coding sequences (Pruitt *et al.* 2009) and their corresponding orthologs from each method. For each possible ortholog (defined by human target sequence and species of origin), we examine whether sequence identity to human is at least five percentage points greater for one method *vs.* another. We otherwise consider the two methods to be tied. By this metric, one method significantly outperforms another 38−45% of the time. Importantly, no method uniformly outperforms all others, underlining the complementarity of the chosen algorithms. For each method, distributions of percent identity and relative performance by species are presented in Figure S1 and Figure S2,

#### Combining multiple sequence alignments with MOSAIC:

It is well-known in theory (Wolpert and Macready 1997) and in practice (Van Der Laan and Gruber 2010) that the comparative performance of competing statistical inference algorithms often varies by context. Rather than search for a single best algorithm, researchers have sought to integrate a variety of methods in order to reap the benefits of methodological complementarity (Van Der Laan *et al.* 2007; Rokach 2009; Chandrasekaran and Jordan 2013). As might be expected, the gains yielded by this approach generally scale with the quality of the individual methods integrated, the number of methods included, and, importantly, the diversity of the comprised algorithms (Kuncheva and Whitaker 2003).

Having observed the complementarity between OD methods, we sought to develop a structure for the automatic integration of methodologically distinct OD methods such as those described above. We term this framework MOSAIC, or **M**ultiple **O**rthologous **S**equence **A**nalysis and **I**ntegration by **C**luster optimization. MOSAIC allows for the flexible integration of diverse OD methods through the application of standard or user-defined metrics of sequence similarity and ortholog cluster quality. By the use of the specified similarity metrics, clusters of proposed orthologs are built. These orthologs are then adopted or rejected to optimize cluster completeness and quality (*e.g.*, similarity to a reference sequence or average pairwise similarity).

**Figure 3:**
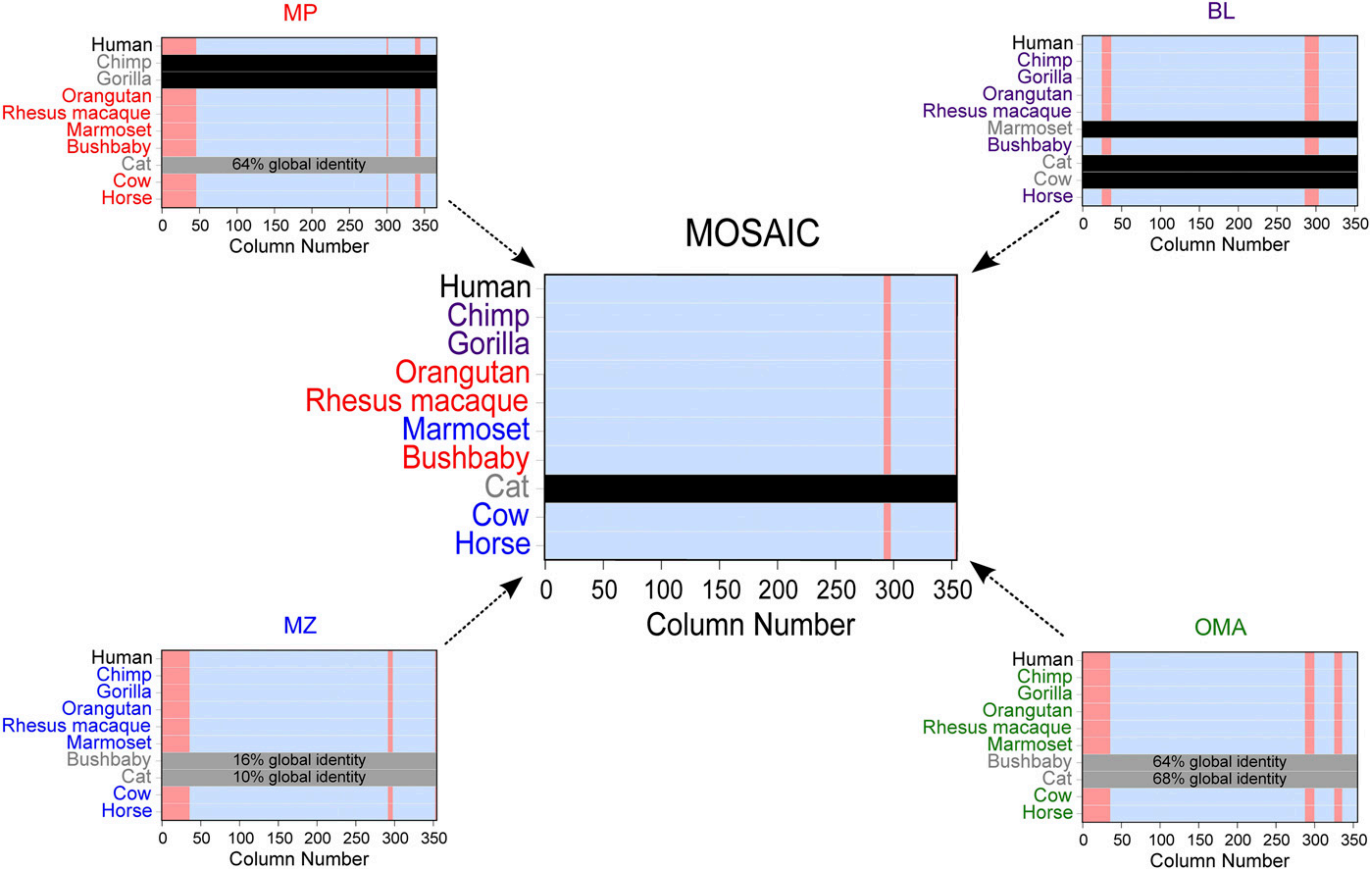
Illustration of integration process for carbonic anhydrase 12. MSA columns that are aligned to below 95% confidence are displayed in red. Orthologs that were not returned for a given species are denoted with a horizontal black bar. Those that were filtered using pre-integration sequence identity cutoffs are indicated with gray bars with the global percent identity from pairwise alignment to human included. Species name label colors denote the species of origin for orthologs in the MOSAIC alignment.

Having presented a schematic of the algorithm itself in Figure 1, we provide in Figure 3 a view of example inputs and output MSAs. These are illustrations of real alignments for carbonic anhydrase 12, an enzyme critical to a number of biological functions, including the formation of bone, saliva, and gastric acid (Pruitt *et al.* 2014). MSA columns that are aligned to below 95% confidence are displayed in red and masked from the analysis. Orthologs that were not returned for a given species are denoted with a horizontal black bar. Those that were filtered using pre-integration sequence identity cutoffs (see the section *Materials and Methods*) are indicated with gray bars. Sequence identity is measured based on pairwise realignment to the human sequence. Note that, just as when employing a single ortholog detection method, this filtering step is critical to guaranteeing alignment quality.

### Method integration increases the number of included sequences

**Figure 4:**
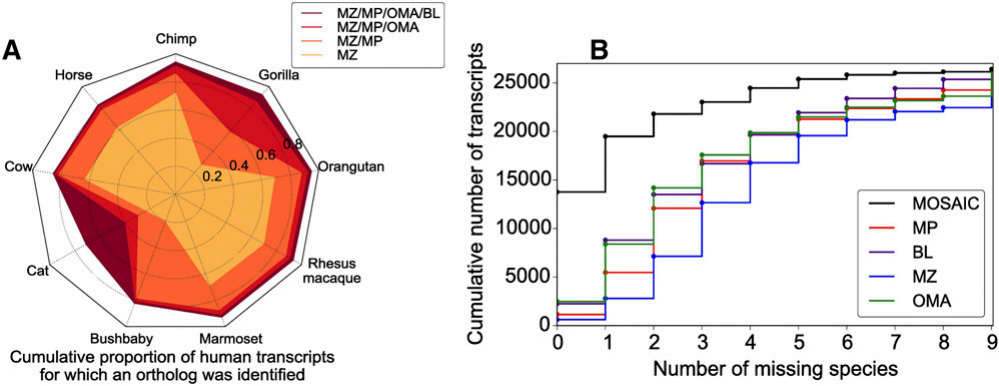
OD power and the effect of pooling methods (A) The cumulative proportion of human transcripts for which an ortholog was detected, stratified by species. Envelopes illustrate results from pooling an increasing number of methods. (B) The cumulative number of human transcripts as a function of the maximum number of missing species allowed.

The gains afforded by MOSAIC vary by species and increase with the number of methods that are included (Figure 4A). When all four component methods are included, MOSAIC more than quintuples the number of alignments for which all species are present (Figure 4B). We observe in Figure 4A that the largest improvements are seen for gorilla, bushbaby, and cat. Importantly, orthologs for each of these three species are rescued by different methods (OMA for gorilla, MultiParanoid for bushbaby, and Blat for cat. See Figure S3 for further details). In the sections that follow, we will demonstrate that MOSAIC captures these additional sequences while simultaneously improving functional-, phylogenetic-, and sequence identity-based measures of ortholog quality.

### MOSAIC improves sequence identity

MOSAIC achieves massive gains in the number of retrieved orthologs while slightly improving average levels of sequence identity. Although MOSAIC directly optimizes sequence identity, this result is noncircular for two reasons. First, average levels of sequence identity could be reduced by preferentially adding sequences from the lower end of the sequence identity distribution. This result would be consistent with a scenario in which most methods correctly inferred that a gene was deleted on a particular lineage. Second, MOSAIC optimizes sequence identity measured from pairwise global alignments. In the validation phase, we calculated this metric in the context of the full MSA. That is, we do not realign to the human sequence in a pairwise fashion as we do in the optimization phase. Rather, we measure sequence identity based on the alignment specified within the full MSA. We believe this choice is sensible because it is the quality of the MSA that is of primary importance to many downstream phylogenetic inference tasks. In addition, this approach allows us to indirectly incorporate information about intra-cluster similarity. As an MSA incorporates increasingly divergent sequences, performance relative to pairwise alignments is expected to progressively degrade.

### MOSAIC improves functional concordance

We employed profile HMMs from the Protein Families Database A (PfamA) (Punta *et al.* 2012) and HMMER3 (Eddy 2011) to ascertain putative functional concordance between proposed orthologs and the human consensus coding sequences of interest. PfamA builds HMMs via curated alignments of small numbers of representative members from each protein family. It is important to note that not all PfamA HMMs are functionally validated. In cases where experimental validation is unavailable, these HMMs provide a family-specific scoring function that yields information not contained in naïve sequence identity measures.

**Figure 5:**
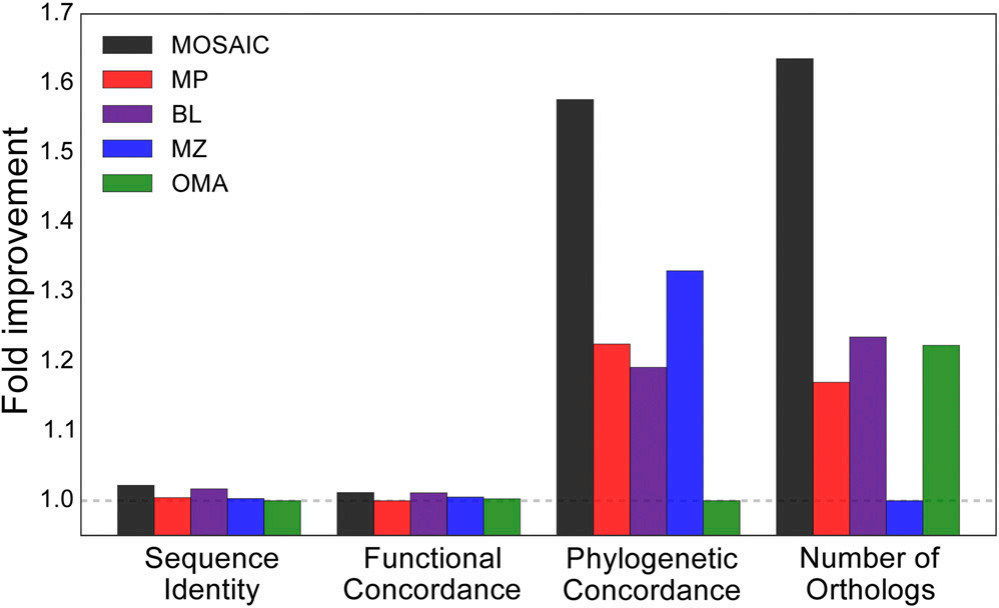
MOSAIC improves alignment quality. We show the fold improvement of each method over the worst performing method in four categories: sequence identity, functional concordance, phylogenetic concordance, and number of orthologs detected.

With HMMER3, we queried protein sequences against all PfamA protein family profiles, annotating each protein according to its top protein family hit. This allowed for an ascertainment of functional concordance that is more comprehensive than relying on gene-by-gene annotation across species, while retaining many of the advantages of manual curation where it exists. This assessment reveals that, for the set of orthologous sequences proposed by all methods, MOSAIC provides levels of functional concordance that are slightly better than the best performing component method (Figure 5). Gains are particularly large for gorilla, bushbaby, and cat orthologs (Figure S4).

### MOSAIC improves phylogenetic concordance

Phylogenetic concordance was ascertained by calculating the normalized, unweighted Robinson-Foulds distance (Robinson and Foulds 1981) between gene trees and the established species tree (Altenhoff and Dessimoz 2009b). This metric is equal to the sum of the number of splits in one tree that are not present in the other, scaled by the total number of splits present across the two trees. Accordingly, larger Robinson-Foulds distances correspond to worse agreement between gene and species trees. On a gene-by-gene basis, this metric should be interpreted with caution, since post-speciation admixture and incomplete lineage sorting can lead to true discordance between the species tree and the phylogenetic history of a particular gene (Maddison and Knowles 2006). At greater levels of divergence, loss of signal and homoplasy may similarly confound the analysis in some cases. However, at the level of the genome, higher concordance between gene trees and the known speciation process strongly suggests a relative improvement in OD.

Figure 5 presents a comparison of genome-wide phylogenetic concordance (see the section *Materials and Methods* for details on this metric). MultiZ performs the best of any individual method, likely due to its utilization of syntenic information. Surprisingly, OMA, the OD method that incorporates phylogenetic tree information, exhibits the worst performance according to this tree-based metric. MOSAIC, on the other hand, provides significant performance gain over all component methods, including a 59% increase in phylogenetic concordance compared to OMA.

### Increased ortholog quality leads to more conservation *and* positively selected sites

Having demonstrated an increase in ortholog quality using tree-, function-, and similarity-based measures of quality, we next sought to assess the influence of increased alignment quality on estimated levels of selection. To assess gene-level conservation, we applied PAML (Yang 2007) with automated likelihood-based model selection. To ascertain site-level positive selection, we used SLR, a method shown to have a greater power and a lower false positive rate than PAML’s popular Bayes Empirical Bayes method (Massingham and Goldman 2005).

Because varying numbers of sequences can sway evolutionary estimates in unpredictable ways due to, *e.g.*, inhomogeneous levels of selection across organisms, we assessed the performance of MOSAIC relative to each method by matching the species present in each alignment. We refer to this approach as MOSAIC_matched_. In the case of both PAML and SLR, synonymous substitution rates in coding DNA are used as a background against which to test for changes in rates of non-synonymous substitution. We compared our performance to that of metaPhOrs, an OD integration method that works on tree-based methods only. Since the metaPhOrs database provides only protein sequences for its alignments, no evolutionary comparison with this method was possible given the available data. However, we demonstrate in Figure S6 that MOSAIC outperforms metaPhOrs according to the metrics presented in Figure 3, despite integrating nearly half the number of OD methods in this example. An analysis with a matching number of OD methods was not possible because metaPhOrs is available only as a pre-calculated database.

**Figure 6:**
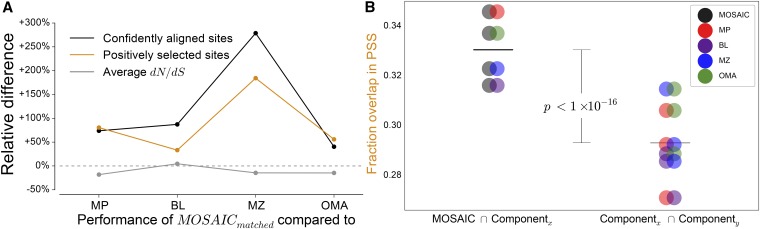
A comparison of evolutionary estimates. (A) The relative difference of MOSAIC_matched_ *vs.* each component method for: (1) the number of positively selected sites, (2) the number of confidently aligned sites, and for reference, (3) the average level of conservation across all alignments. (B) The agreement between positively selected sites (1) between MOSAIC and component methods, and (2) among component methods. Fractional overlap values are plotted as Venn diagrams to illustrate the two methods being compared.

In Figure 6B, we see that MOSAIC leads to greater gene-level conservation (lower dN/dS) compared with every method except Blat, for which the difference was not statistically significant. Full distributions of dN/dS for each method are presented in Figure S7. Despite greater levels of conservation, MOSAIC was able to detect ~30–180% more positively selected sites than any of its component methods. This was not due to an increase in the inferred rate of positive selection. Rather, most of this increase in power was attributable to the fact that more sites were aligned to high confidence and therefore included in the analysis. This step of filtering for alignment quality is important because site-wise estimates of positive selection are highly sensitive to short poorly aligned regions (Jordan and Goldman 2012).

To investigate the quality of the positively selected sites detected by MOSAIC, we assessed concordance with and between component methods. For a pair of method, we measure overlap by dividing the total size of the intersection between positively selected sites by the total size of the union. These results are shown in Figure 6B. We observe that the minimum overlap between MOSAIC and a component method (MOSAIC/Blat) is still better than the best overlap between component methods (MultiZ/OMA). Averaging over comparisons, we find the improvement in concordance with *vs.* between component methods is statistically significant beyond computational precision (*P* < 1e-16).

### Understanding MOSAIC’s improvements in performance

As we have mentioned, ensemble-based inference frameworks have been shown to operate effectively in a wide variety of statistical contexts. For the methods examined here, MOSAIC may provide increased MSA quality for a variety of reasons, not all of which are related to the performance of the component algorithms. For example, the ability to specify a custom scoring function for pairwise similarity gives MOSAIC an advantage over component methods. For example, BLAST-based scoring such as that used by MultiParanoid may in some cases reward substitutions over than sequence identity. This is the result of building substitution matrices on libraries of sequences that are much more highly diverged than those within mammalian proteomes.

The quality of a genome assembly also plays an important role. For methods like MultiZ, improperly assembled genomic segments may provide misleading information about orthologous relationships. Similarly, naïve methods such as BLAT will miss proteins whose exons are spread across unassembled genomic segments. Other methods such as MultiParanoid and OMA may be robust to this effect since they draw from more sophisticated proteome predictions. However, the stringent and sometimes arbitrary filtering required for proteome prediction cause these methods to miss sequences that can be found using BLAT’s six-frame genomic translation.

### Better alignments may yield new insights into human evolution

**Figure 7:**
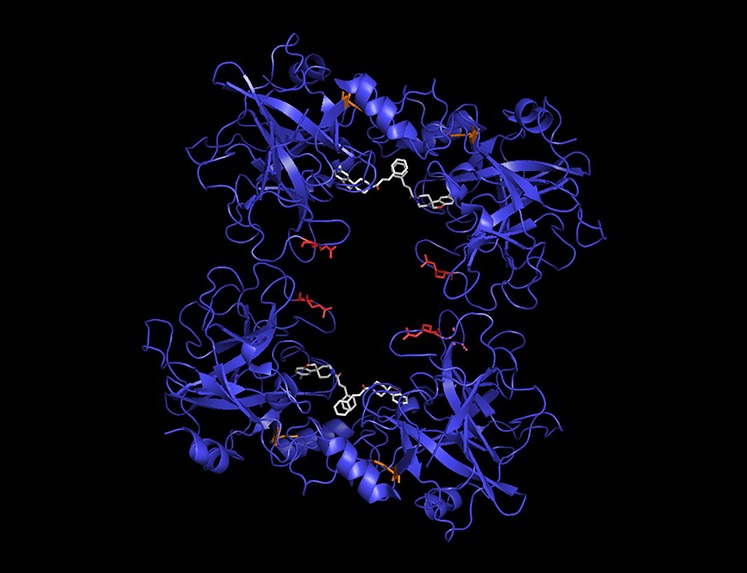
Example: a MOSAIC-specific PSS in Tryptase Alpha/Beta 1 (TPSAB1). The tetrameric TPSAB1 structure is shown with positively selected sites highlighted. The site detected by component methods and by MOSAIC is colored orange, whereas the MOSAIC-specific PSS is featured in red. A bound inhibitor (white) pinpoints the active site of the enzyme.

We next sought to examine the biological significance of some of the positively selected sites identified uniquely by MOSAIC. This led us to Tryptase Alpha/Beta 1 (TPSAB1), a tetrameric serine protease that has been implicated in the pathogenesis of asthma (Taira *et al.* 2002; Cui *et al.* 2014), heart disease (Bot *et al.* 2014), inflammatory bowel disease (Hamilton *et al.* 2011), and other disorders with allergic and/or inflammatory components (Sommerhoff and Schaschke 2007). Shown in Figure 7 is the three-dimensional structure of a TPSAB1 tetramer with inhibitor (white) bound (Costanzo *et al.* 2008). In orange, distal to the active site, is the positively selected residue detected by component methods and by MOSAIC. Note that positive selection at this location is active only outside of the great apes, with a fixed lysine observed in human, chimp, gorilla, and orangutan (Figure S9 and Figure S10).

In red, directly within the proteolytic pore, is the site identified by MOSAIC as positively selected. This residue is a positively charged arginine in humans. This would be expected to modify the electrostatics of ligand binding. In chimp, we instead observe a kink-inducing proline. We might anticipate this change to have a large steric effect, possibly allowing the inward-facing unstructured loop to act as a more rigid lid closing over top of the substrate, or as a modifier of subunit contacts. Importantly, these changes occurred repeatedly in mammals. Proline is observed at this position in rhesus macaque and marmoset. Arginine, on the other hand, is present in gorilla and horse (Figure S9 and Figure S10). In orangutan, we observe a histidine: another positively charged amino acid.

Throughout this examination, we must be cognizant that tryptases evolved rapidly during primate evolution (Trivedi *et al.* 2007). The expansion of this gene family can itself be viewed as an example of positive selection. However, the presence of several paralogs creates the risk of inappropriately aligning pseudo-orthologous sequences that have evolved to serve divergent functions. Given the challenges, this case study provides an excellent opportunity to compare the high-throughput performance of MOSAIC to that of manually curated alignments.

As a first step, we showed that each proposal ortholog was a best bidirectional hit to TPSAB1 (Table S1). Next, we compared our sequences to those retrieved manually by Trivedi *et al.* (2007). Although we notice a few minor discrepancies between the two sets of alignments (see Figure S9 *vs.* Figure S11, reproduced from Trivedi *et al.* 2007), these differences do not alter our conclusion of human-relevant positive selection at the highlighted site in the proteolytic core of TPSAB1. Illustrations of component alignments from each method are shown in Figure S8.

In this paper we have introduced a novel algorithm, MOSAIC, which is capable of integrating an arbitrary number of methodologically diverse ortholog detection methods. We have demonstrated that MOSAIC provides large increases in power relative to its component methods, while simultaneously maintaining or improving functional-, phylogenetic-, and sequence identity-based measures of ortholog quality. Further, given the same number of species, MOSAIC alignments include more columns aligned with high confidence. This translates to higher levels of estimated conservation, and simultaneously, a greatly increased number of positively selected sites detected. Moreover, MOSAIC’s positively selected sites agree better with those from component methods than component results do with each other. This suggests that not only does MOSAIC detect more positively selected sites—these sites are more reproducible and are detected due to an increase in alignment quality. Finally, we illustrated the significance of this increase in power by highlighting a positively selected site near the active site of the tryptase TPSAB1. Given the role of this enzyme in asthma and other allergic and inflammatory disorders, we feel that this case study is worthy of experimental follow-up.

In summary, MOSAIC provides the unique flexibility to incorporate any OD method that may be available now or in the future. It can therefore capture the entire swath of methodologic diversity, thereby improving OD performance, and allowing researchers to take advantage of methodological gains in a variety of areas of OD research. In addition, it provides the flexibility to adapt scoring and optimization procedures to the set of species under study. In future work, it will be interesting to ascertain how optimal procedures vary between species sets that have differing mean levels of divergence and markedly different patterns of evolution. For example, mammals and prokaryotes will likely have distinct optimal parameter values within MOSAIC. This tool is available a python package that can be installed using easy_install bio-mosaic from the command-line. MOSAIC alignments, source code, and full documentation are available at http://pythonhosted.org/bio-MOSAIC. 

## 

## Supplementary Material

Supporting Information
