# Supplementary material for: Rock, Paper, Scissors: Harnessing Complementarity in Ortholog Detection Methods Improves Comparative Genomic Inference
Source: G3 (Bethesda). 2015 Feb 23;5(4):629–38. doi: 10.1534/g3.115.017095 (PMC4390578; doi:10.1534/g3.115.017095)
Supplement: Supporting Information [file supp_5_4_629__index.html]

Rock, Paper, Scissors: Harnessing Complementarity in Ortholog Detection Methods Improves Comparative Genomic Inference — Supporting Information 

# Rock, Paper, Scissors: Harnessing Complementarity in Ortholog Detection Methods Improves Comparative Genomic Inference

## Supporting Information for Maher and Hernandez, 2015

**Files in this Data Supplement:**

- Supporting Information - Figures S1-S11 and Table S1 (PDF, 12 MB)
- Figure S1 - Distributions of percent identity relative to the highest scoring ortholog, stratified by species. (PDF, 358 KB)
- Figure S2 - The effect of method integration on sequence identity. (PDF, 273 KB)
- Figure S3 - The cumulative proportion of transcripts for which an ortholog is identified. (PDF, 156 KB)
- Figure S4 - The rate of concordance between functional annotations for proposal orthologs and human transcripts. (PDF, 202 KB)
- Figure S5 - The cumulative proportion of human transcripts as a function of the maximum allowable Robinson-Foulds distance between the gene tree and the species tree. (PDF, 157 KB)
- Figure S6 - A comparison between MOSAIC and metaPhOrs. (PDF, 238 KB)
- Figure S7 - The distribution of gene-level conservation (measured by dN/dS) for each component method versus MOSAICmatched. (PDF, 141 KB)
- Figure S8 - A representation of the alignments returned by each method for TPSAB1. (PDF, 318 KB)
- Figure S9 - The MOSAIC alignment of TPSAB1. (PDF, 10 MB)
- Figure S10 - The *Gorilla gorilla* sequence that is orthologous to TPSAB1. (PDF, 85 KB)
- Table S1 - SwissProt database BLAST results for each of the putative orthologs of TPSAB1. (PDF, 71 KB)
- Figure S11 - Manually derived alignments of TPSAB1, reproduced from Trivedi *et al.* 2007. (PDF, 581 KB)
