# Supplementary material for: Rock, Paper, Scissors: Harnessing Complementarity in Ortholog Detection Methods Improves Comparative Genomic Inference
Source: G3 (Bethesda). 2015 Feb 23;5(4):629–38. doi: 10.1534/g3.115.017095 (PMC4390578; doi:10.1534/g3.115.017095)
Supplement: Supporting Information [file supp_g3.115.017095_017095SI.pdf]

G3 · Genes | Genomes | Genetics

Rock, Paper, Scissors: harnessing complementarity in ortholog detection methods improves comparative genomic inference.

M. Cyrus Maher[*, 1] and Ryan D. Hernandez [§,†,‡, 1]

[*]Department of Epidemiology and Biostatistics, University of California, San Francisco [§]Department of Bioengineering and Therapeutic Sciences, [‡]Institute for Human Genetics, [†]Institute for Quantitative Biosciences (QB3), University of California, San Francisco, 1700 4th Street San Francisco, California 94158

[1]Corresponding author. E-mail: ryan.hernandez@ucsf.edu

DOI: 10.1534/g3.115.017095

Figure S1 demonstrates that, for each species, MOSAIC retrieves a much larger number of sequences than any method alone, while maintaining levels of percent identity comparable to those of the best performing method. It should be noted here that in our current examples, MOSAIC is designed to optimize the metric of sequence identity to human. Indeed, for a given putative ortholog, MOSAIC is guaranteed to improve or maintain percent identity compared to its constituent methods. Counter-intuitively, this provides no assurance that MOSAIC will provide gains in *average* levels of percent identity. For example, average levels of percent identity could decrease if MOSAIC ensures the inclusion of a greater number of species by pulling in poorly scoring sequences that were initially filtered out by the majority of component methods. However in Figure S1, we see that this is not the case.

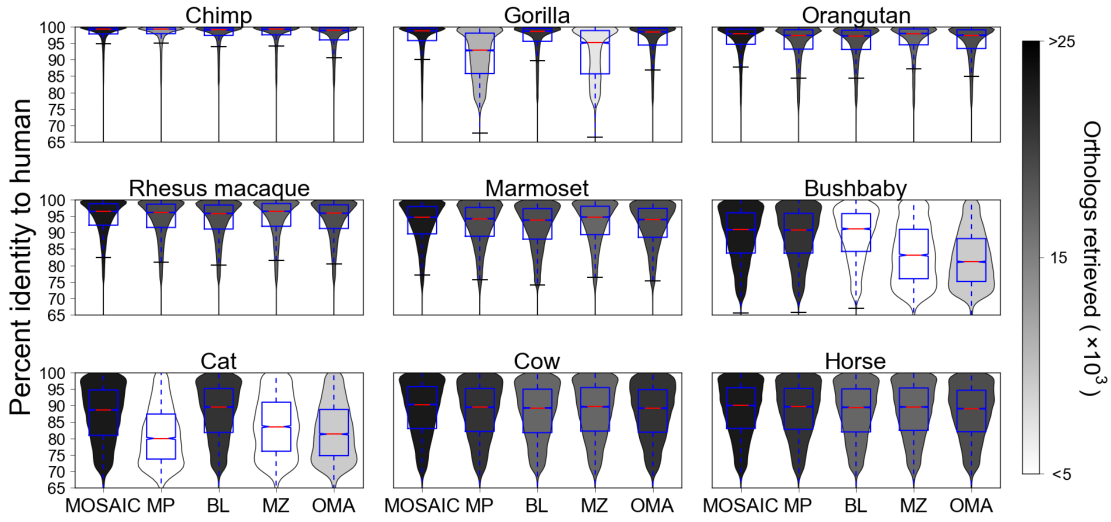

Figure S1. Distributions of percent identity relative to the highest scoring ortholog, stratified by species. *This plot demonstrates how each method's performance compares to the best method. Each data point is a putative ortholog from a given species. Distributions are summarized by violinplots with boxplots overlaid*

We next evaluated percent identity to human for each ortholog proposed by each method relative to the highest scoring ortholog from all methods. Figure S2 demonstrates that relative performance is species-specific. In particular, we note that the performance disparities across methods are much more pronounced for gorilla, bushbaby, and cat, both in terms of the number and quality of obtained orthologs.

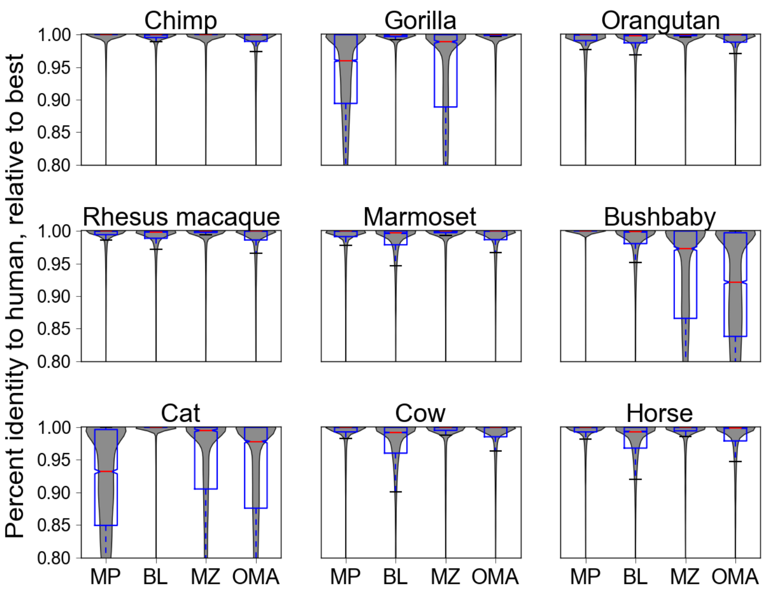

Figure S2. ***The effect of method integration on sequence identity.*** *A comparison of the overall distributions of percent identity to human for MOSAIC and its component methods. Smoothed distributions underlying the boxplots are shaded according to the number of human transcripts for which an ortholog was proposed. White denotes 5000 sequences or less. Darker shades signify increasingly larger numbers of detected orthologs.*

Examining each OD method in detail yields some hypotheses about the origin of these differences in performance. Errors in proteome prediction, both in terms of false-positives and false-negatives, are likely to have large effects on both MultiParanoid and OMA. Meanwhile, spurious syntenic information is expected to compromise the integrity of ortholog predictions produced by MultiZ. Finally, the lack of an assembled genome for bushbaby may negatively impact the quality of BLAT due to the segmentation of exon sets across multiple unordered scaffolds.

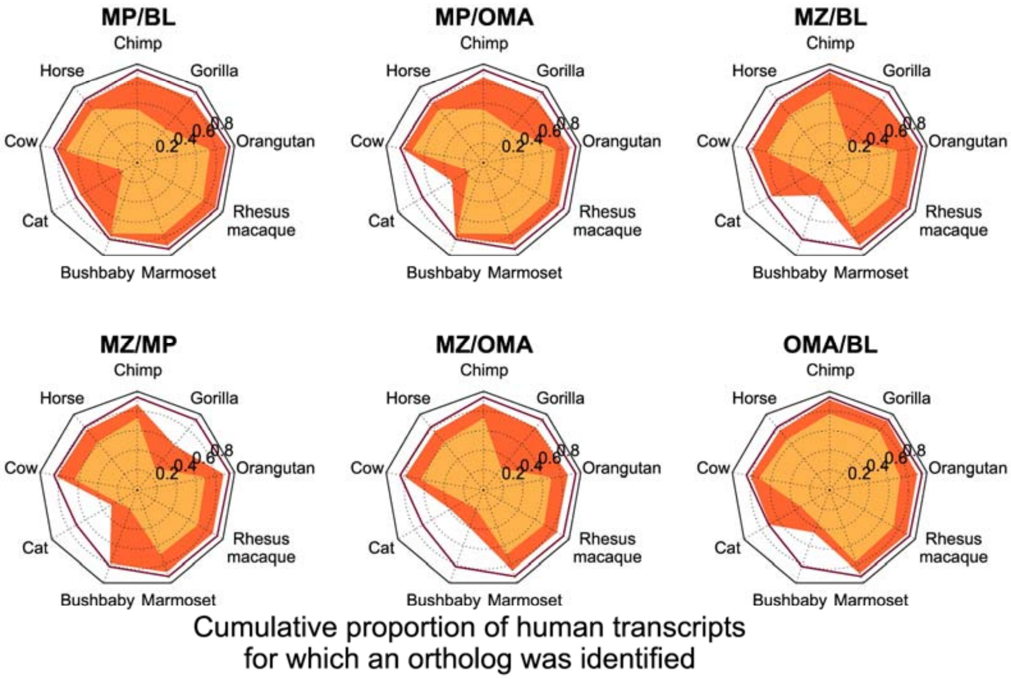

Figure S3. The cumulative proportion of transcripts for which an ortholog is identified. We show how all pairs of methods perform in retrieving orthologs for each species.

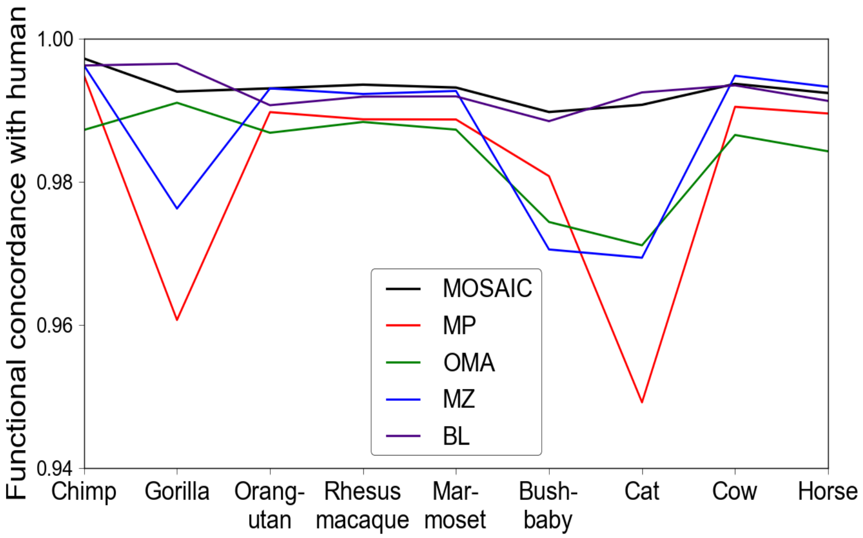

Figure S4. The rate of concordance between functional annotations for proposal orthologs and human transcripts.

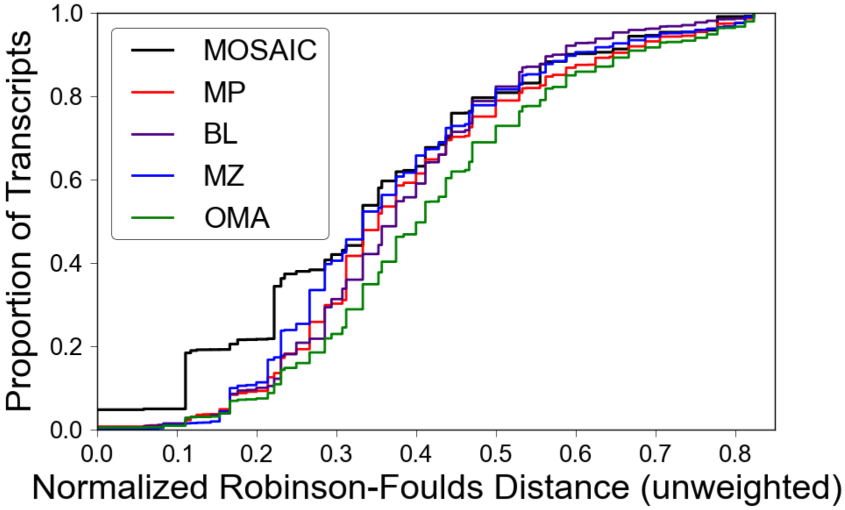

Figure S5. The cumulative proportion of human transcripts as a function of the maximum allowable Robinson-Foulds distance between the gene tree and the species tree.

Figure S5 presents the cumulative proportion of alignments included as a function of the maximum allowable RF distance. Multiz is seen to perform the best of any individual method, likely due to its utilization of syntenic information. Surprisingly, the tree-based OD method, OMA, is seen to be the worst performing method according to this tree-based metric. Combining all methods using MOSAIC leads to a strong enrichment of highly concordant gene trees, while providing performance that is competitive with all component methods at more permissive RF distance cutoffs.

Comparison to a related method

We have shown that MOSAIC provides a large increase in the number of detected orthologs relative to its component methods, while simultaneously maintaining or improving functional-, phylogenetic-, and sequence identity-based measures of ortholog quality. Next, we sought to compare this method of OD integration to the only alternative of which we are aware: metaPhOrs (Pryszcz et al. 2011). Using an approach based on tree overlap, metaPhOrs integrates ortholog predictions using phylogenetic trees from seven databases: PhylomeDB, Ensembl, TreeFam, EggNOG, OrthoMCL, COG, and Fungal Orthogroups.

While MOSAIC is able to integrate an arbitrary number of OD methods of any time, metaPhOrs can only integrate tree-based methods. Since only pre-computed metaPhOrs data is available, we can also only examine the results of integrating the seven methods named above. This is then skewed comparison because MOSAIC only integrates four methods. Nevertheless, we compared MOSAIC and metaPhOrs based on the number of retrieved orthologs, average differences in sequence identity, and comparative levels of functional and phylogenetic concordance. We observe that MOSAIC provides large increases in the number of retrieved orthologs, while providing slight improvements in sequence identity for those cases where proposal orthologs are available from both methods (fig. S6). For the cases where MOSAIC predicted an ortholog but metaPhOrs did not, we examined the level of sequence identity in these sequences compared to the species-specific average returned by metaPhOrs. We find that these additional sequences display levels of sequence identity comparable to those provided by metaPhOrs. Finally, we observe that MOSAIC yields a slight increase in functional concordance, as well as a 40% increase in tree concordance, measured as the area under the curve below an RF distance of 0.5. A 0.5 threshold was chosen because there is little differentiation between methods after this point.

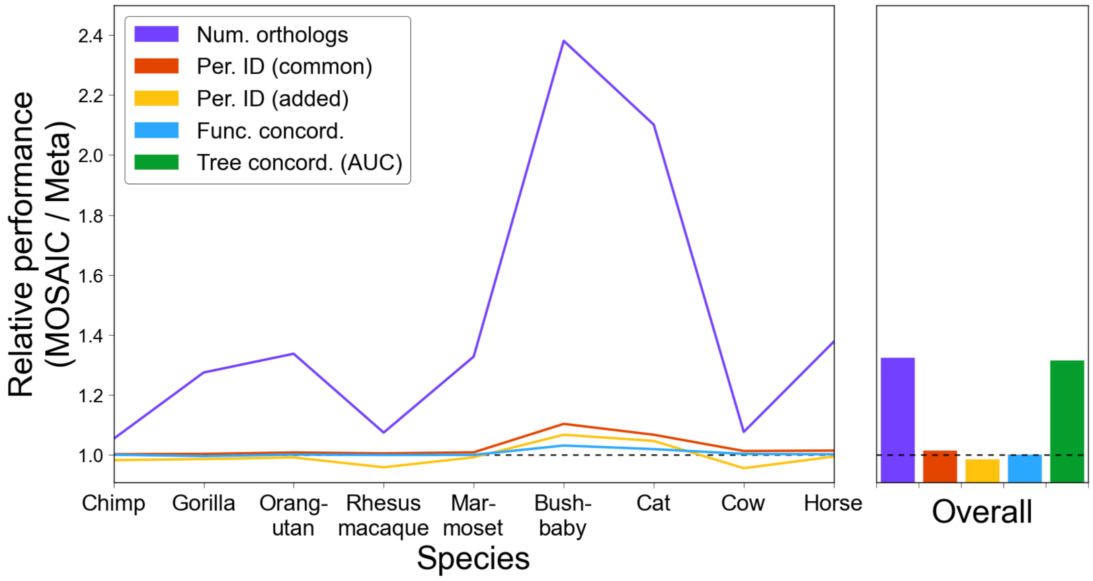

Figure S6. A comparison between MOSAIC and metaPhOrs. *The relative performance between MOSAIC and metaPhOrs according to five metrics: 1.) the number of orthologs detected (purple); 2.) the percent identity to human for orthologs present in both (red); 3.) the percent identity to human for orthologs unique to MOSAIC compared to metaPhOrs species-specific average (yellow); 4.) rate of functional concordance between proposal orthologs and human transcripts (blue); and 5.) concordance between gene and species trees, as measured by a normalized, unweighted Robinson-Foulds distance (green). A.) The breakdown of relative performance by species. B.) Relative performance averaged across species. Scale is matched to panel A. Note that tree concordance is only included in panel B because it is calculated based upon full sequence alignments.*

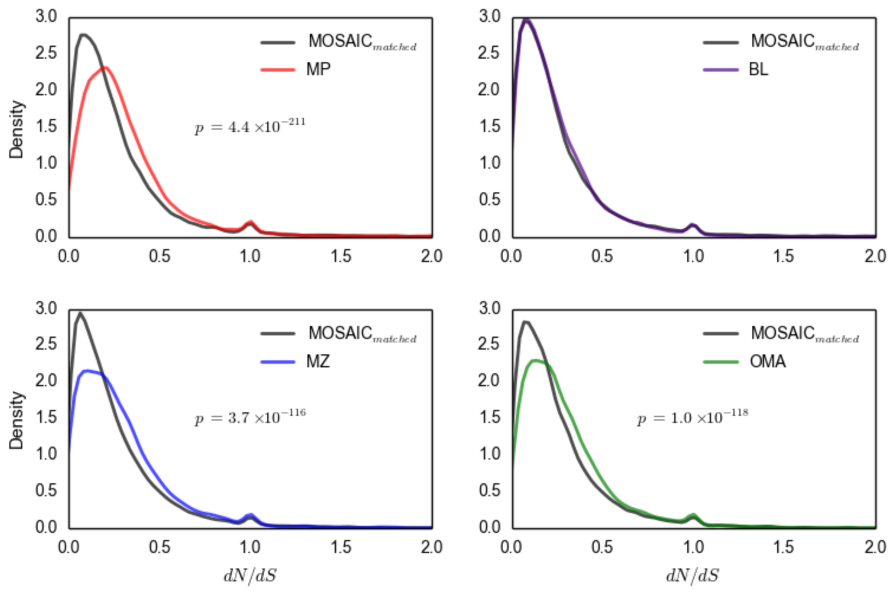

Figure S7. ***The distribution of gene-level conservation (measured by dN/dS) for each component method versus MOSAIC$_{matched}$.***

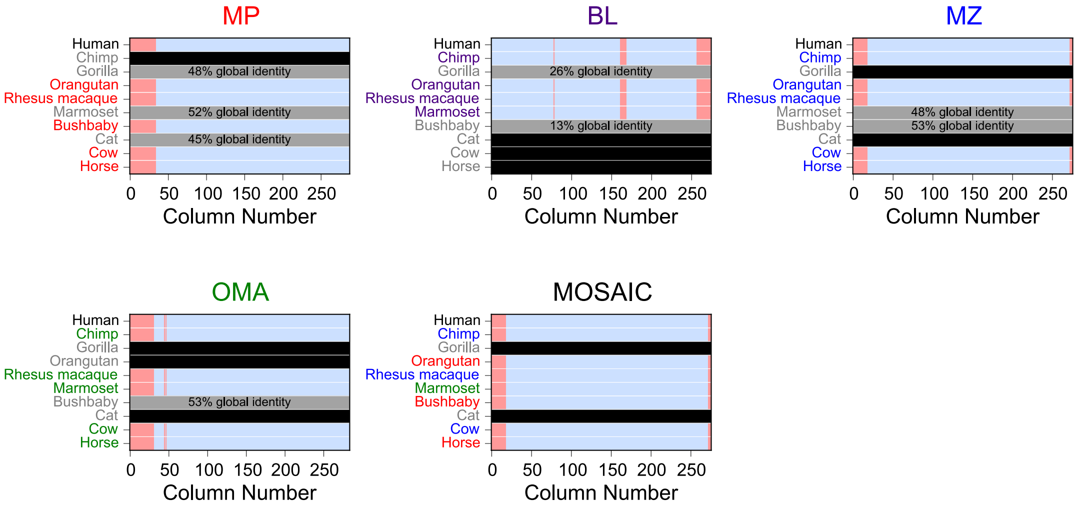

Figure S8. A representation of the alignments returned by each method for TPSAB1.

```
CCDS10431.1     MLNLLLLALPVLASRAYAAPAPGQALQRVGIVGGQEAPRSKWPWQVSLRVHGPYWMHFCGGSLIHPQWVLTAAHCVGPDV 80
Pan [[multiz]]  MLSLLLLALPILASPAYAAPAPGQALQRAGIVGGQEAPRSKWPWQVSLRVRDRYWMHFCGGSLIHPQWVLTAAHCVGPDF 80
Pon [[inpara]]  MLSLLLLALPVLASPAYAAPAPGQALQRVGIVGGQEAPRSKWPWQVSLRVHGQYWMHFCGGSLIHPQWVLTAAHCVGPDV 80
Mac [[multiz]]  MLNLLLLALPVLVSPAHAAPAPGQALQRVGIVGGQEAPRSKWPWQVSLRLHGQYWMHFCGGSLIHPQWVLTAAHCVGPDV 80
Cal [[OMA]]     MLSLLLLVLPVLVSLAHSAPAPGQALPRAGIVGGQEAPGSRWPWQVSLRFHSQFWMHFCGGSLIHPQWVLTAAHCLGPDV 80
Oto [[inpara]]  MLSLLVLALPILGSRVHAAPAPGQASERAGIVGGQEAPESKWPWQVSLRQHTHFWMHICGGSLIHPQWVLTAAHCVGPEV 80
Bos [[multiz]]  MLHL--LALALLLSLVSAAPAPGQALQRAGIVGGQEAPGSRWPWQVSLRVSHQYWRHHCGGSLIHPQWVLTAAHCVGPEV 78
Equ [[inpara]]  MPNLLVLALALLVNLGHAAPAPGQALEREGIVGGQEASGSKWPWQVSLRKNTEYWKHFCGGSLIHPQWVLTAAHCVGPDI 80

CCDS10431.1     KDLAALRVQLREQHLYYQDQLLPVSRIIVHPQFYTAQIGADIALLELEEPVNVSSHVHTVTLPPASETFPPGMPCWVTGW 160
Pan [[multiz]]  KDLATLRVQLQEQHLYYQDQLLPVSRIIVHPQFYIIQTGADIALLELEEPVNVSSRVHTVTLPPASETFPPGMPCWVTGW 160
Pon [[inpara]]  KDLAALRVQLREQHLYYQDQLLPVGRIIVHPQFYTAQTGADIALLELEEPVNISSHVHTVTLPPASETFPPGMPCWVTGW 160
Mac [[multiz]]  KDLADLRVQLREQHLYYQDQLLPVSRIIVHPQFYAVQIGADIALLELEEPVNVSSHVHTVTLPPASETFPPGTPCWVTGW 160
Cal [[OMA]]     MDLANLRVQLREQHLYYKDRLLPVSRLIVHPQFYIVQTGADIALLELEEPVNVSSHVRTVTLPPASETFPAGTPCWVTGW 160
Oto [[inpara]]  QDLADFRVQLREQHLYYHDKLLPVSRIIPHPGFYMATTGADIALLELEEPVNISHSVHTITLPPASETFPPGTPCWVTGW 160
Bos [[multiz]]  HGPSYFRVQLREQHLYYQDQLLPISRIIPHPNYYSVENGADIALLELDEPVSISCHVQPVTLPPESETFPPGTQCWVTGW 158
Equ [[inpara]]  EDFRDIRVQLREQHLYYRDQLLPVSRILPHPYYYTVENGADIALLELQDPVNISSHVQVVTLPPASETFPPGTPCWVTGW 160

CCDS10431.1     GDVDNDERLPPPFPLKQVKVPIMENHICDAKYHLGAYTGDDVRIVRDDMLCAGNTRRDSCQGDSGGPLVCKVNGTWLQAG 240
Pan [[multiz]]  GDVDNDEPLPPPFPLKQVKVPIMENHICDAKYHLGAYTGDDVRIIRDDMLCAGNTRRDSCQGDSGGPLVCKVNGTWLQAG 240
Pon [[inpara]]  GDVDNDEHLPPPFPLKQVKVPIMENHICDAKYHLGLYTGDDVRIVRDDMLCAGNSRRDSCQGDSGGPLVCKVNGTWLQAG 240
Mac [[multiz]]  GDVDNDVPLPPPFPLKQVKVPIMENHICDAKYHSGLYTGDDVRIIRDDMLCAGNSRRDTCQGDSGGPLVCKVNGTWLQAG 240
Cal [[OMA]]     GDVNTGEPLPPPFPLKQVKVPIVENQVCDMKYHAGLYTGDAVHIVRDDMLCAGNSRRDSCQGDSGGPLVCKVNDTWLQAG 240
Oto [[inpara]]  GDVDNDVGLPPPFPLKQVKVPIVENHICDAKYHMGLYTGDNVHIVGDNMLCAGNTRKDSCQGDSGGPLVCKVNGTWLQAG 240
Bos [[multiz]]  GNVDNGRRLPPPFPLKQVKVPVVENSVCDRKYHSGLSTGDNVPIVQEDNLCAGDSGRDSCQGDSGGPLVCKVNGTWLQAG 238
Equ [[inpara]]  GDVDNGVSLPPPFPLKEVKVPIVENSVCDRKYHTGVSTGDNIRIVQADMLCAGNRRHDSCQGDSGGPLVCKVKGTWLQAG 240

CCDS10431.1     VVSWGEGCAQPNRPGIYTRVTYYLDWIHHYVPKKP- 275
Pan [[multiz]]  VVSWDEGCAQPNRPGIYTRVTYYLDWIHHYVPKKHX 276
Pon [[inpara]]  VVSWGEGCAQPNRPGIYTRVTYYLDWIHRYVPKKP- 275
Mac [[multiz]]  VVSWDEGCAQPYRPGIYTRITYYLDWIHRYVPEKPX 276
Cal [[OMA]]     VVSWGEGCALPNRPGIYTRVTYYLDWIHQYVPKKP- 275
Oto [[inpara]]  VVSWGDGCAQPNRPGIYTRVTHYLDWIHHYVPKEP- 275
Bos [[multiz]]  VVSWGDGCAKPNRPGIYTRVTSYLDWIHQYVPQGPX 274
Equ [[inpara]]  VVSWANSCAQPNRPGIYTRVTYYLDWIYQYVPKDS- 275
```

Figure S9. The MOSAIC alignment of TPSAB1. *The MOSAIC-specific positively selected site is illustrated with the red arrow, while the site detected by several methods, including MOSAIC, is indicated in gold.*

>gi|146150402|gb|ABQ02500.1|:1-275 beta 1 tryptase [Gorilla gorilla]

MLNLLLLALPVLASPAYAAPAPGQALQRAGIVGGQEAPRSKWPWQVSLRVRGQYWMHFCGGSLIHPQWVLTAAHCVGPDVKDLAALRVQLRE
QHLYYQDQLLPVSRIIVHPQFYTAQIGADIALLELEEPVNVSSHVHTVTLPPASETFPPGMPCWVTGWGDVDNDE**R**LPPPFPLKQVKVPIMENHIC
DAKYH**L**GAYTGDNVRIVRDDMLCAGNTRRDSCQGDSGGPLVCKVNGTWLQAGVVSWGEGCAQPNRPGIYTRVTYYLDWIHHYVPKKP

Figure S10. The *Gorilla gorilla* sequence that is orthologous to TPSAB1. *A* Gorilla gorilla gorilla *sequence was not present, presumably due to genome quality issues. For the* Gorilla gorilla *sequence, we highlight the residues of the positively selected sites indicated in Figure S9.*

Table S1. SwissProt database BLAST results for each of the putative orthologs of TPSAB1.

Query species	Best match	% ID	% Similarity	Alignment length	Mismatches	E-value
Chimp	TPSAB1	94	95	262	15	0
Orangutan	TPSAB1	96	97	275	10	0
Rhesus Mac.	TPSAB1	92	95	263	21	2.0E-180
Marmoset	TPSAB1	85	90	262	39	3.0E-166
Bushbaby	TPSAB1	84	90	263	41	5.0E-167
Cow	TPSAB1	77	86	262	60	1.0E-148
Horse	TPSAB1	79	87	258	54	2.0E-153

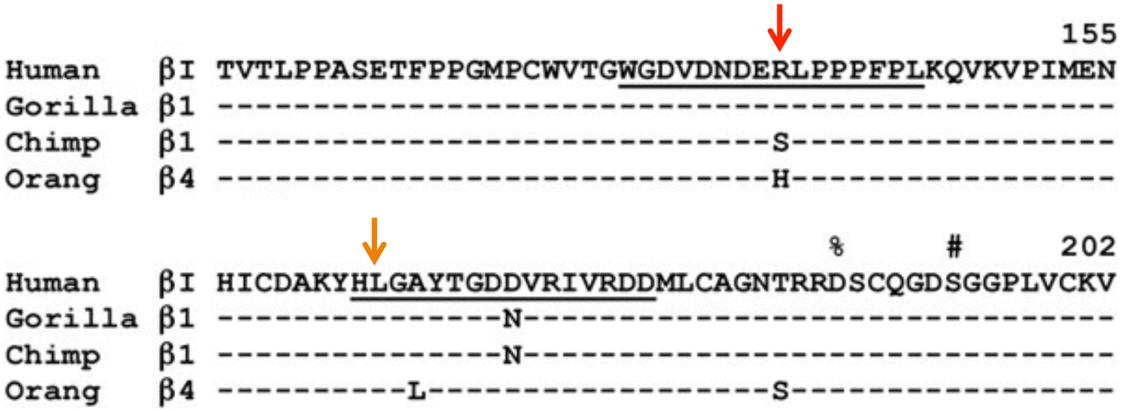

Figure S11. Manually derived alignments of TPSAB1, reproduced from Trivedi et al. 2007. *As above, The MOSAIC-specific positively selected site is illustrated with the red arrow, while the site detected by several methods, including MOSAIC, is indicated in gold.*
