# Supplementary material for: Rock, Paper, Scissors: Harnessing Complementarity in Ortholog Detection Methods Improves Comparative Genomic Inference
Source: G3 (Bethesda). 2015 Feb 23;5(4):629–38. doi: 10.1534/g3.115.017095 (PMC4390578; doi:10.1534/g3.115.017095)
Supplement: Supporting Information [file supp_g3.115.017095_FigureS1.pdf]

Figure S1 demonstrates that, for each species, MOSAIC retrieves a much larger number of sequences than any method alone, while maintaining levels of percent identity comparable to those of the best performing method. It should be noted here that in our current examples, MOSAIC is designed to optimize the metric of sequence identity to human. Indeed, for a given putative ortholog, MOSAIC is guaranteed to improve or maintain percent identity compared to its constituent methods. Counter-intuitively, this provides no assurance that MOSAIC will provide gains in *average* levels of percent identity. For example, average levels of percent identity could decrease if MOSAIC ensures the inclusion of a greater number of species by pulling in poorly scoring sequences that were initially filtered out by the majority of component methods. However in Figure S1, we see that this is not the case.

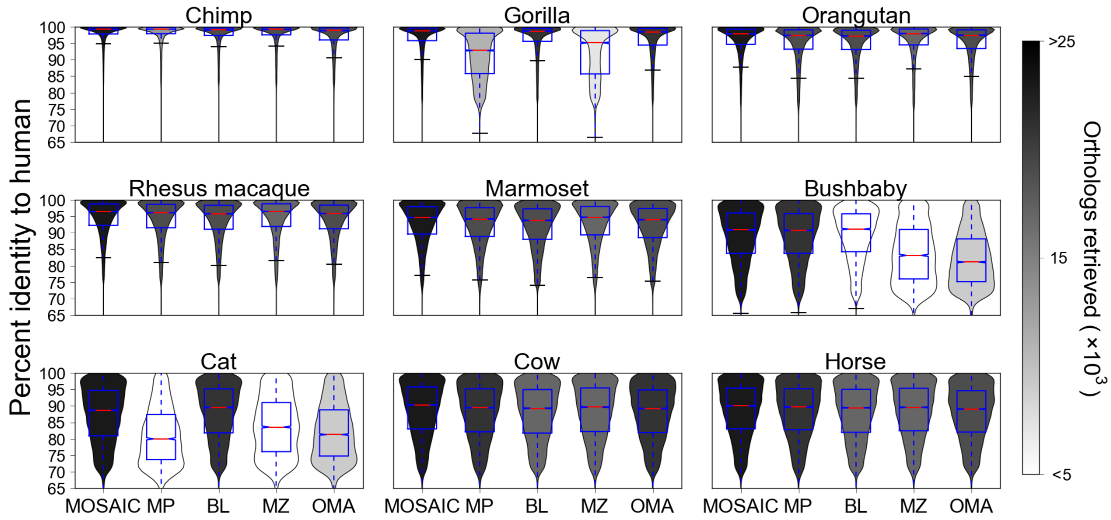

Figure S1. Distributions of percent identity relative to the highest scoring ortholog, stratified by species. *This plot demonstrates how each method's performance compares to the best method. Each data point is a putative ortholog from a given species. Distributions are summarized by violinplots with boxplots overlaid*

We next evaluated percent identity to human for each ortholog proposed by each method relative to the highest scoring ortholog from all methods. Figure S2 demonstrates that relative performance is species-specific. In particular, we note that the performance disparities across methods are much more pronounced for gorilla, bushbaby, and cat, both in terms of the number and quality of obtained orthologs.
