# Supplementary material for: Rock, Paper, Scissors: Harnessing Complementarity in Ortholog Detection Methods Improves Comparative Genomic Inference
Source: G3 (Bethesda). 2015 Feb 23;5(4):629–38. doi: 10.1534/g3.115.017095 (PMC4390578; doi:10.1534/g3.115.017095)
Supplement: Supporting Information [file supp_g3.115.017095_FigureS2.pdf]

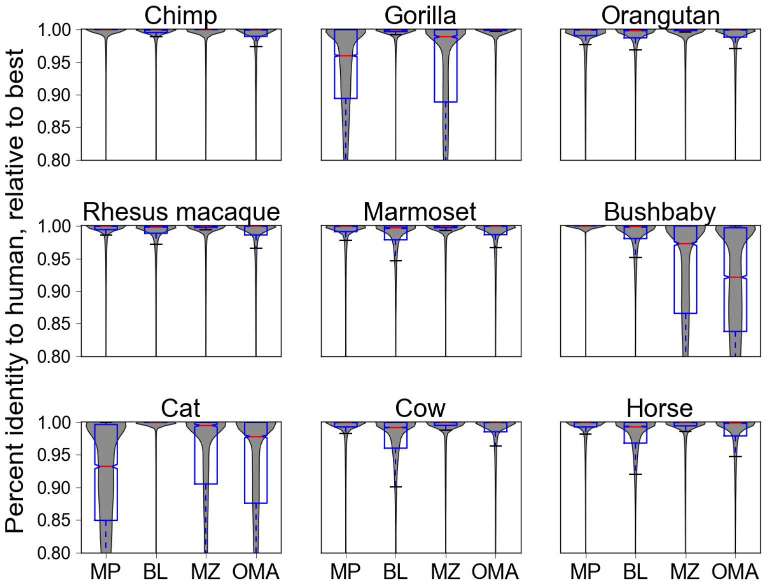

Figure S2. ***The effect of method integration on sequence identity.*** *A comparison of the overall distributions of percent identity to human for MOSAIC and its component methods. Smoothed distributions underlying the boxplots are shaded according to the number of human transcripts for which an ortholog was proposed. White denotes 5000 sequences or less. Darker shades signify increasingly larger numbers of detected orthologs.*

Examining each OD method in detail yields some hypotheses about the origin of these differences in performance. Errors in proteome prediction, both in terms of false-positives and false-negatives, are likely to have large effects on both MultiParanoid and OMA. Meanwhile, spurious syntenic information is expected to compromise the integrity of ortholog predictions produced by MultiZ. Finally, the lack of an assembled genome for bushbaby may negatively impact the quality of BLAT due to the segmentation of exon sets across multiple unordered scaffolds.
