# Supplementary material for: Rock, Paper, Scissors: Harnessing Complementarity in Ortholog Detection Methods Improves Comparative Genomic Inference
Source: G3 (Bethesda). 2015 Feb 23;5(4):629–38. doi: 10.1534/g3.115.017095 (PMC4390578; doi:10.1534/g3.115.017095)
Supplement: Supporting Information [file supp_g3.115.017095_FigureS3.pdf]

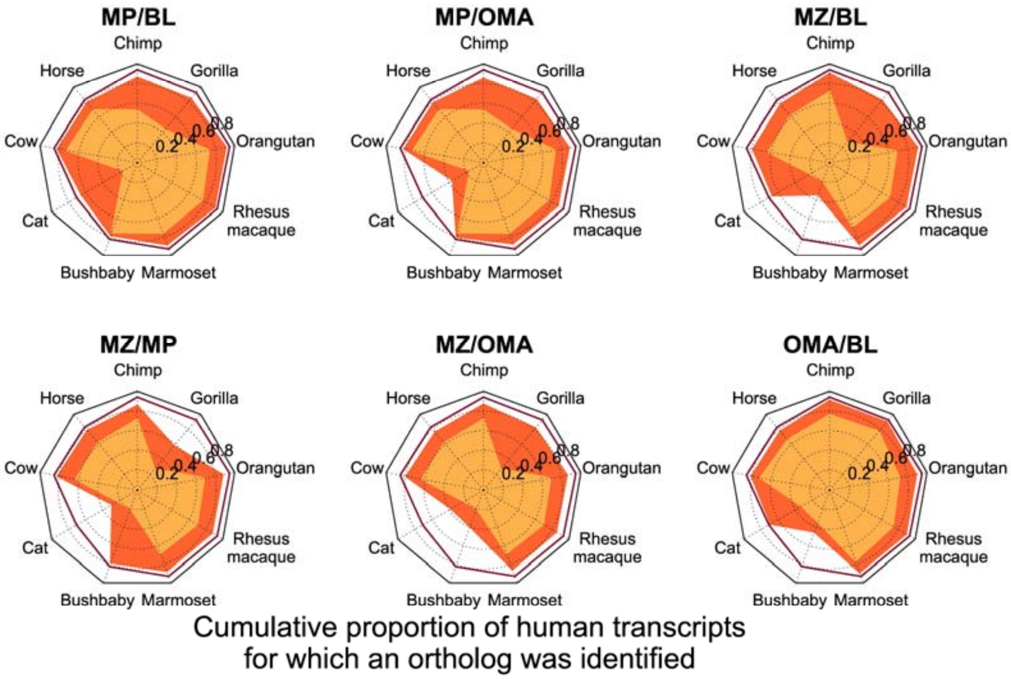

Figure S3. The cumulative proportion of transcripts for which an ortholog is identified. We show how all pairs of methods perform in retrieving orthologs for each species.
