# Supplementary material for: Rock, Paper, Scissors: Harnessing Complementarity in Ortholog Detection Methods Improves Comparative Genomic Inference
Source: G3 (Bethesda). 2015 Feb 23;5(4):629–38. doi: 10.1534/g3.115.017095 (PMC4390578; doi:10.1534/g3.115.017095)
Supplement: Supporting Information [file supp_g3.115.017095_FigureS5.pdf]

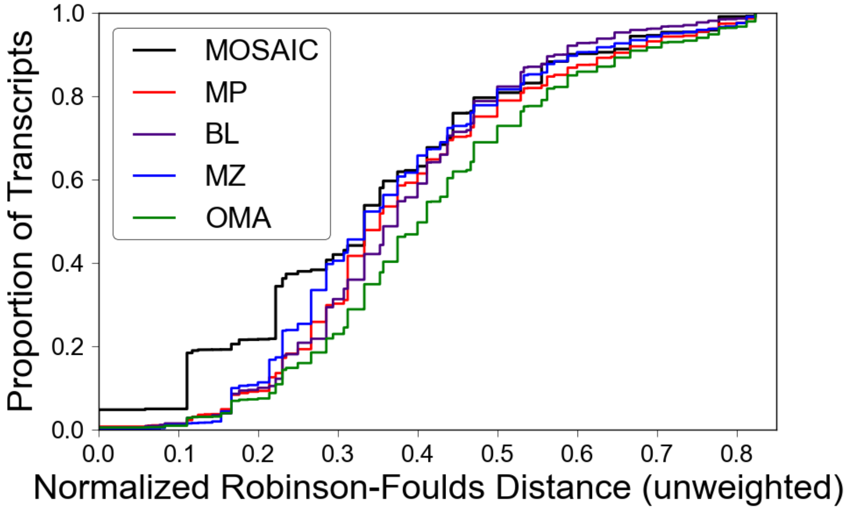

Figure S5. The cumulative proportion of human transcripts as a function of the maximum allowable Robinson-Foulds distance between the gene tree and the species tree.

Figure S5 presents the cumulative proportion of alignments included as a function of the maximum allowable RF distance. Multiz is seen to perform the best of any individual method, likely due to its utilization of syntenic information. Surprisingly, the tree-based OD method, OMA, is seen to be the worst performing method according to this tree-based metric. Combining all methods using MOSAIC leads to a strong enrichment of highly concordant gene trees, while providing performance that is competitive with all component methods at more permissive RF distance cutoffs.
