# Supplementary material for: Rock, Paper, Scissors: Harnessing Complementarity in Ortholog Detection Methods Improves Comparative Genomic Inference
Source: G3 (Bethesda). 2015 Feb 23;5(4):629–38. doi: 10.1534/g3.115.017095 (PMC4390578; doi:10.1534/g3.115.017095)
Supplement: Supporting Information [file supp_g3.115.017095_FigureS6.pdf]

Comparison to a related method

We have shown that MOSAIC provides a large increase in the number of detected orthologs relative to its component methods, while simultaneously maintaining or improving functional-, phylogenetic-, and sequence identity-based measures of ortholog quality. Next, we sought to compare this method of OD integration to the only alternative of which we are aware: metaPhOrs (Pryszcz et al. 2011). Using an approach based on tree overlap, metaPhOrs integrates ortholog predictions using phylogenetic trees from seven databases: PhylomeDB, Ensembl, TreeFam, EggNOG, OrthoMCL, COG, and Fungal Orthogroups.

While MOSAIC is able to integrate an arbitrary number of OD methods of any time, metaPhOrs can only integrate tree-based methods. Since only pre-computed metaPhOrs data is available, we can also only examine the results of integrating the seven methods named above. This is then skewed comparison because MOSAIC only integrates four methods. Nevertheless, we compared MOSAIC and metaPhOrs based on the number of retrieved orthologs, average differences in sequence identity, and comparative levels of functional and phylogenetic concordance. We observe that MOSAIC provides large increases in the number of retrieved orthologs, while providing slight improvements in sequence identity for those cases where proposal orthologs are available from both methods (fig. S6). For the cases where MOSAIC predicted an ortholog but metaPhOrs did not, we examined the level of sequence identity in these sequences compared to the species-specific average returned by metaPhOrs. We find that these additional sequences display levels of sequence identity comparable to those provided by metaPhOrs. Finally, we observe that MOSAIC yields a slight increase in functional concordance, as well as a 40% increase in tree concordance, measured as the area under the curve below an RF distance of 0.5. A 0.5 threshold was chosen because there is little differentiation between methods after this point.

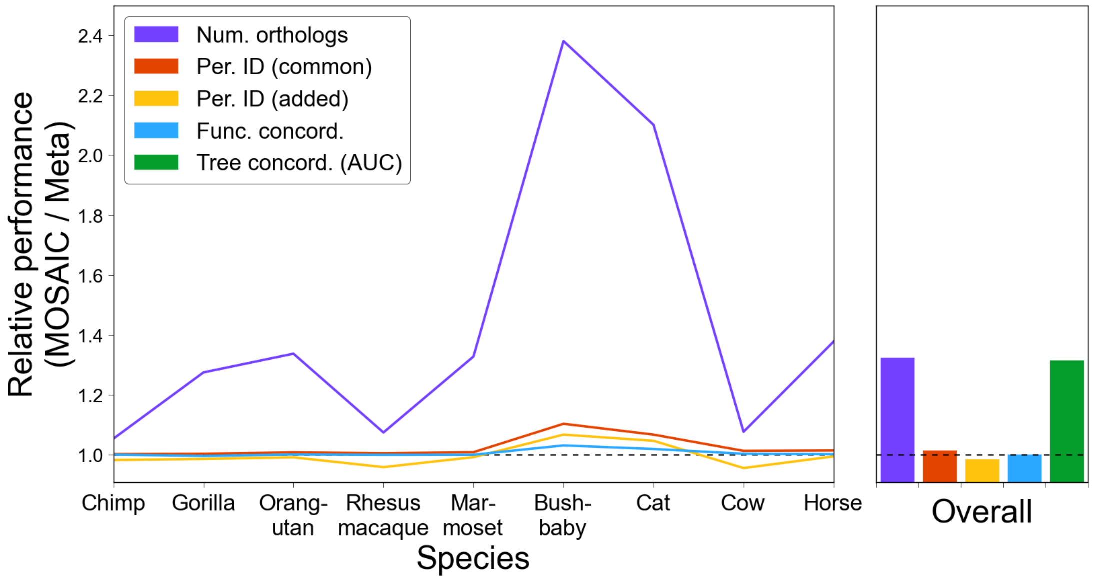

Figure S6. A comparison between MOSAIC and metaPhOrs. *The relative performance between MOSAIC and metaPhOrs according to five metrics: 1.) the number of orthologs detected (purple); 2.) the percent identity to human for orthologs present in both (red); 3.) the percent identity to human for orthologs unique to MOSAIC compared to metaPhOrs species-specific average (yellow); 4.) rate of functional concordance between proposal orthologs and human transcripts (blue); and 5.) concordance between gene and species trees, as measured by a normalized, unweighted Robinson-Foulds distance (green). A.) The breakdown of relative performance by species. B.) Relative performance averaged across species. Scale is matched to panel A. Note that tree concordance is only included in panel B because it is calculated based upon full sequence alignments.*
