# Supplementary figures and images for: Rock, Paper, Scissors: Harnessing Complementarity in Ortholog Detection Methods Improves Comparative Genomic Inference
Source: G3 (Bethesda). 2015 Feb 23;5(4):629–38. doi: 10.1534/g3.115.017095 (PMC4390578; doi:10.1534/g3.115.017095)

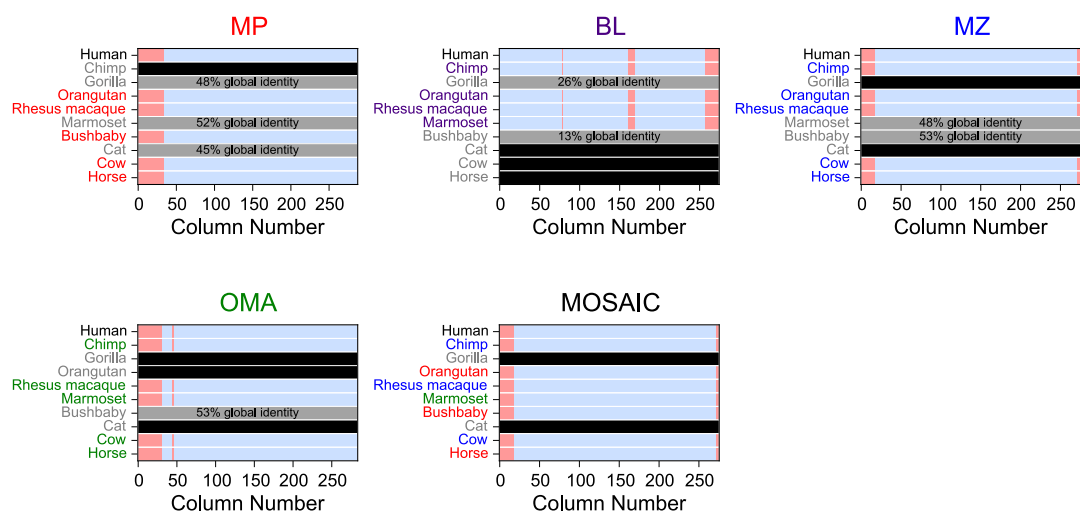

Figure S8. A representation of the alignments returned by each method for TPSAB1.

Supplement: Supporting Information [file supp_g3.115.017095_FigureS8.pdf]
