# Supplementary material for: Rock, Paper, Scissors: Harnessing Complementarity in Ortholog Detection Methods Improves Comparative Genomic Inference
Source: G3 (Bethesda). 2015 Feb 23;5(4):629–38. doi: 10.1534/g3.115.017095 (PMC4390578; doi:10.1534/g3.115.017095)
Supplement: Supporting Information [file supp_g3.115.017095_FigureS9.pdf]

```
CCDS10431.1     MLNLLLLALPVLASRAYAAPAPGQALQRVGIVGGQEAPRSKWPWQVSLRVHGPYWMHFCGGSLIHPQWVLTAAHCVGPDV 80
Pan [[multiz]]  MLSLLLLALPILASPAYAAPAPGQALQRAGIVGGQEAPRSKWPWQVSLRVRDRYWMHFCGGSLIHPQWVLTAAHCVGPDF 80
Pon [[inpara]]  MLSLLLLALPVLASPAYAAPAPGQALQRVGIVGGQEAPRSKWPWQVSLRVHGQYWMHFCGGSLIHPQWVLTAAHCVGPDV 80
Mac [[multiz]]  MLNLLLLALPVLVSPAHAAPAPGQALQRVGIVGGQEAPRSKWPWQVSLRLHGQYWMHFCGGSLIHPQWVLTAAHCVGPDV 80
Cal [[OMA]]     MLSLLLLVLPVLVSLAHSAPAPGQALPRAGIVGGQEAPGSRWPWQVSLRFHSQFWMHFCGGSLIHPQWVLTAAHCLGPDV 80
Oto [[inpara]]  MLSLLVLALPILGSRVHAAPAPGQASERAGIVGGQEAPESKWPWQVSLRQHTHFWMHICGGSLIHPQWVLTAAHCVGPEV 80
Bos [[multiz]]  MLHL--LALALLLSLVSAAPAPGQALQRAGIVGGQEAPGSRWPWQVSLRVSHQYWRHHCGGSLIHPQWVLTAAHCVGPEV 78
Equ [[inpara]]  MPNLLVLALALLVNLGHAAPAPGQALEREGIVGGQEASGSKWPWQVSLRKNTEYWKHFCGGSLIHPQWVLTAAHCVGPDI 80

CCDS10431.1     KDLAALRVQLREQHLYYQDQLLPVSRIIVHPQFYTAQIGADIALLELEEPVNVSSHVHTVTLPPASETFPPGMPCWVTGW 160
Pan [[multiz]]  KDLATLRVQLQEQHLYYQDQLLPVSRIIVHPQFYIIQTGADIALLELEEPVNVSSRVHTVTLPPASETFPPGMPCWVTGW 160
Pon [[inpara]]  KDLAALRVQLREQHLYYQDQLLPVGRIIVHPQFYTAQTGADIALLELEEPVNISSHVHTVTLPPASETFPPGMPCWVTGW 160
Mac [[multiz]]  KDLADLRVQLREQHLYYQDQLLPVSRIIVHPQFYAVQIGADIALLELEEPVNVSSHVHTVTLPPASETFPPGTPCWVTGW 160
Cal [[OMA]]     MDLANLRVQLREQHLYYKDRLLPVSRLIVHPQFYIVQTGADIALLELEEPVNVSSHVRTVTLPPASETFPAGTPCWVTGW 160
Oto [[inpara]]  QDLADFRVQLREQHLYYHDKLLPVSRIIPHPGFYMATTGADIALLELEEPVNISHSVHTITLPPASETFPPGTPCWVTGW 160
Bos [[multiz]]  HGPSYFRVQLREQHLYYQDQLLPISRIIPHPNYYSVENGADIALLELDEPVSISCHVQPVTLPPESETFPPGTQCWVTGW 158
Equ [[inpara]]  EDFRDIRVQLREQHLYYRDQLLPVSRILPHPYYYTVENGADIALLELQDPVNISSHVQVVTLPPASETFPPGTPCWVTGW 160

CCDS10431.1     GDVDNDERLPPPFPLKQVKVPIMENHICDAKYHLGAYTGDDVRIVRDDMLCAGNTRRDSCQGDSGGPLVCKVNGTWLQAG 240
Pan [[multiz]]  GDVDNDEPLPPPFPLKQVKVPIMENHICDAKYHLGAYTGDDVRIIRDDMLCAGNTRRDSCQGDSGGPLVCKVNGTWLQAG 240
Pon [[inpara]]  GDVDNDEHLPPPFPLKQVKVPIMENHICDAKYHLGLYTGDDVRIVRDDMLCAGNSRRDSCQGDSGGPLVCKVNGTWLQAG 240
Mac [[multiz]]  GDVDNDVPLPPPFPLKQVKVPIMENHICDAKYHSGLYTGDDVRIIRDDMLCAGNSRRDTCQGDSGGPLVCKVNGTWLQAG 240
Cal [[OMA]]     GDVNTGEPLPPPFPLKQVKVPIVENQVCDMKYHAGLYTGDAVHIVRDDMLCAGNSRRDSCQGDSGGPLVCKVNDTWLQAG 240
Oto [[inpara]]  GDVDNDVGLPPPFPLKQVKVPIVENHICDAKYHMGLYTGDNVHIVGDNMLCAGNTRKDSCQGDSGGPLVCKVNGTWLQAG 240
Bos [[multiz]]  GNVDNGRRLPPPFPLKQVKVPVVENSVCDRKYHSGLSTGDNVPIVQEDNLCAGDSGRDSCQGDSGGPLVCKVNGTWLQAG 238
Equ [[inpara]]  GDVDNGVSLPPPFPLKEVKVPIVENSVCDRKYHTGVSTGDNIRIVQADMLCAGNRRHDSCQGDSGGPLVCKVKGTWLQAG 240

CCDS10431.1     VVSWGEGCAQPNRPGIYTRVTYYLDWIHHYVPKKP- 275
Pan [[multiz]]  VVSWDEGCAQPNRPGIYTRVTYYLDWIHHYVPKKHX 276
Pon [[inpara]]  VVSWGEGCAQPNRPGIYTRVTYYLDWIHRYVPKKP- 275
Mac [[multiz]]  VVSWDEGCAQPYRPGIYTRITYYLDWIHRYVPEKPX 276
Cal [[OMA]]     VVSWGEGCALPNRPGIYTRVTYYLDWIHQYVPKKP- 275
Oto [[inpara]]  VVSWGDGCAQPNRPGIYTRVTHYLDWIHHYVPKEP- 275
Bos [[multiz]]  VVSWGDGCAKPNRPGIYTRVTSYLDWIHQYVPQGPX 274
Equ [[inpara]]  VVSWANSCAQPNRPGIYTRVTYYLDWIYQYVPKDS- 275
```

Figure S9. The MOSAIC alignment of TPSAB1. *The MOSAIC-specific positively selected site is illustrated with the red arrow, while the site detected by several methods, including MOSAIC, is indicated in gold.*
