# Supplementary material for: Rock, Paper, Scissors: Harnessing Complementarity in Ortholog Detection Methods Improves Comparative Genomic Inference
Source: G3 (Bethesda). 2015 Feb 23;5(4):629–38. doi: 10.1534/g3.115.017095 (PMC4390578; doi:10.1534/g3.115.017095)
Supplement: Supporting Information [file supp_g3.115.017095_FigureS10.pdf]

>gi|146150402|gb|ABQ02500.1|:1-275 beta 1 tryptase [Gorilla gorilla]

MLNLLLLALPVLASPAYAAPAPGQALQRAGIVGGQEAPRSKWPWQVSLRVRGQYWMHFCGGSLIHPQWVLTAAHCVGPDVKDLAALRVQLRE
QHLYYQDQLLPVSRIIVHPQFYTAQIGADIALLELEEPVNVSSHVHTVTLPPASETFPPGMPCWVTGWGDVDNDE**R**LPPPFPLKQVKVPIMENHIC
DAKYH**L**GAYTGDNVRIVRDDMLCAGNTRRDSCQGDSGGPLVCKVNGTWLQAGVVSWGEGCAQPNRPGIYTRVTYYLDWIHHYVPKKP

Figure S10. The *Gorilla gorilla* sequence that is orthologous to TPSAB1. *A* Gorilla gorilla gorilla *sequence was not present, presumably due to genome quality issues. For the* Gorilla gorilla *sequence, we highlight the residues of the positively selected sites indicated in Figure S9.*
