# Supplementary material for: Rock, Paper, Scissors: Harnessing Complementarity in Ortholog Detection Methods Improves Comparative Genomic Inference
Source: G3 (Bethesda). 2015 Feb 23;5(4):629–38. doi: 10.1534/g3.115.017095 (PMC4390578; doi:10.1534/g3.115.017095)
Supplement: Supporting Information [file supp_g3.115.017095_TableS1.pdf]

Table S1. SwissProt database BLAST results for each of the putative orthologs of TPSAB1.

Query species	Best match	% ID	% Similarity	Alignment length	Mismatches	E-value
Chimp	TPSAB1	94	95	262	15	0
Orangutan	TPSAB1	96	97	275	10	0
Rhesus Mac.	TPSAB1	92	95	263	21	2.0E-180
Marmoset	TPSAB1	85	90	262	39	3.0E-166
Bushbaby	TPSAB1	84	90	263	41	5.0E-167
Cow	TPSAB1	77	86	262	60	1.0E-148
Horse	TPSAB1	79	87	258	54	2.0E-153
