# Supplementary material for: Rock, Paper, Scissors: Harnessing Complementarity in Ortholog Detection Methods Improves Comparative Genomic Inference
Source: G3 (Bethesda). 2015 Feb 23;5(4):629–38. doi: 10.1534/g3.115.017095 (PMC4390578; doi:10.1534/g3.115.017095)
Supplement: Supporting Information [file supp_g3.115.017095_FigureS11.pdf]

```
                                             155
Human   βI TVTLPPASETFPPGMPCWVTGWGDVDNDERLPPPFPLKQVKVPIMEN
Gorilla β1 -----------------------------------------------
Chimp   β1 -----------------------------S-----------------
Orang   β4 -----------------------------H-----------------

                                   %     #     202
Human   βI HICDAKYHLGAYTGDDVRIVRDDMLCAGNTRRDSCQGDSGGPLVCKV
Gorilla β1 ---------------N-------------------------------
Chimp   β1 ---------------N-------------------------------
Orang   β4 ----------L------------------S-----------------
```

Figure S11. Manually derived alignments of TPSAB1, reproduced from Trivedi et al. 2007. *As above, The MOSAIC-specific positively selected site is illustrated with the red arrow, while the site detected by several methods, including MOSAIC, is indicated in gold.*
